# MUR1‐mediated cell‐wall fucosylation is required for freezing tolerance in *Arabidopsis thaliana*

**DOI:** 10.1111/nph.16209

**Published:** 2019-11-07

**Authors:** Paige E. Panter, Olivia Kent, Maeve Dale, Sarah J. Smith, Mark Skipsey, Glenn Thorlby, Ian Cummins, Nathan Ramsay, Rifat A. Begum, Dayan Sanhueza, Stephen C. Fry, Marc R. Knight, Heather Knight

**Affiliations:** ^1^ Department of Biosciences & Durham Centre for Crop Improvement Technology Durham University South Road Durham DH1 3LE UK; ^2^ Scion 49 Sala Street, Private Bag 3020 Rotorua 3046 New Zealand; ^3^ Institute of Molecular Plant Sciences The University of Edinburgh Daniel Rutherford Building, The King’s Buildings, Max Born Crescent Edinburgh EH9 3BF UK

**Keywords:** *Arabidopsis thaliana*, boron, cell wall, freezing tolerance, fucose, pectin, Rhamnogalacturonan II, *sfr8*

## Abstract

- Forward genetic screens play a key role in the identification of genes contributing to plant stress tolerance. Using a screen for freezing sensitivity, we have identified a novel freezing tolerance gene, *SENSITIVE‐TO‐FREEZING8,* in *Arabidopsis thaliana.*
- We identified *SFR8* using recombination‐based mapping and whole‐genome sequencing. As SFR8 was predicted to have an effect on cell wall composition, we used GC‐MS and polyacrylamide gel electrophoresis to measure cell‐wall fucose and boron (B)‐dependent dimerization of the cell‐wall pectic domain rhamnogalacturonan II (RGII) *in planta*. After treatments to promote borate‐bridging of RGII, we assessed freeze‐induced damage in wild‐type and *sfr8* plants by measuring electrolyte leakage from freeze‐thawed leaf discs.
- We mapped the *sfr8* mutation to *MUR1*, a gene encoding the fucose biosynthetic enzyme GDP‐d‐mannose‐4,6‐dehydratase. *sfr8* cell walls exhibited low cell‐wall fucose levels and reduced RGII bridging. Freezing sensitivity of *sfr8* mutants was ameliorated by B supplementation, which can restore RGII dimerization. B transport mutants with reduced RGII dimerization were also freezing‐sensitive.
- Our research identifies a role for the structure and composition of the plant primary cell wall in determining basal plant freezing tolerance and highlights the specific importance of fucosylation, most likely through its effect on the ability of RGII pectin to dimerize.

## Introduction

Plants vary enormously in their capacity to tolerate low temperature. Whilst some tropical species are susceptible to mild chilling, others from cooler parts of the world can tolerate severe sub‐zero temperatures (Burke *et al.*, 1976; Levitt, 1980). Freezing damage manifests itself as dehydration of the cytoplasm and damage to cellular membranes. At very low temperatures, protein denaturation is an additional problem (Thomashow, 1999). During freezing, ice crystals form in the extracellular space because the water in the apoplast has a lower osmotic potential than in the cytoplasm. This leads to very low water potential in the apoplast; therefore, water is drawn out of the protoplast causing dehydration (Thomashow, 1999; Pearce, 2001). Dehydration stress is thus a major component of freeze‐induced damage in plants. Indeed, freezing and drought share many consequences and tolerance of both conditions is effected by some of the same mechanisms, including the upregulation of genes encoding dehydrins (Thomashow, 2010; Nakashima *et al.*, 2014). Upon thawing, if water returns to the cytosol in overabundance or too quickly, the plasma membrane bursts, a phenomenon termed expansion‐induced lysis (EIL) (Uemura *et al.*, 2006), resulting in cell death. Further damage is caused at lower freezing temperatures by the aggregation of membrane lipids into non‐bilayer structures (Gordon‐Kamm & Steponkus, 1984).

The cell wall (CW) provides structural integrity whilst allowing flexibility, extensibility and growth (Cosgrove, 2005). The CW is typically composed of three biochemically distinct components; the primary CW and middle lamella are secreted from the cell first whilst the secondary CW is normally secreted after growth ceases (Popper, 2008). The secondary CW is usually only present in tissues that require structural reinforcement, such as the xylem (Meents *et al.*, 2018). The middle lamella is a pectin‐rich layer shared by adjacent cells and has a number of functions in addition to its role of separating neighbouring cells (Zamil & Geitmann, 2017) whilst the primary CW consists of cellulose microfibrils embedded in a matrix of hemicelluloses and pectins as well as a large number of proteins (Keegstra, 2010). Although much attention has been focussed on the plasma membrane as the major site of damage during and after freezing, the CW can also be significantly affected and may undergo collapse (cytorrhysis) in response to dehydration when extracellular ice forms (Levitt, 1980). Not all plant species undergo CW collapse after freezing and not all CWs exhibit the same growth of ice crystals under similar freezing conditions; it has been suggested that the nature of the CW may be an important determinant of plant freezing tolerance (Rajashekar & Lafta, 1996; Gusta & Wisniewski, 2013). Apoplastic ice‐binding proteins (IBPs) are also important in reducing the growth of ice crystals in the extracellular compartment (Bredow & Walker, 2017).

Some species from temperate parts of the world are capable of cold acclimation (CA), a phenomenon whereby they gain in freezing tolerance (FT) after exposure to low non‐freezing temperatures for a period of days or weeks before the onset of freezing (Thomashow, 1999; Browse & Xin, 2001; Smallwood & Bowles, 2002; Baxter, 2014). During CA, major transcriptional reprogramming occurs (Fowler & Thomashow, 2002; Hannah *et al.*, 2005; Kaplan *et al.*, 2007). This is accompanied by large‐scale changes in the metabolome that bring about increased production of compatible solutes (Stitt & Hurry, 2002; Cook *et al.*, 2004; Kaplan *et al.*, 2007), changes in membrane structure and composition (Steponkus & Lynch, 1989; Uemura & Steponkus, 1994; Uemura *et al.*, 1995), and changes in growth and morphology (Thomashow, 1999). Changes in CW composition (Takahashi *et al.*, 2019), and increased CW thickness and strength also occur (Rajashekar & Lafta, 1996; Stefanowska *et al.*, 1999).

The CBF (C‐Repeat binding factor) transcription factors (*CBF1*, *CBF2* and *CBF3* genes; also known as *DREB1B, DREB1C* and *DREB1A* (Shinwari *et al.*, 1998) control the expression of a large number of cold‐regulated (*COR*) genes encoding proteins with functions associated with CA (Gilmour *et al.*, 2004). The CBFs have been well‐documented elsewhere (for a recent review see (Ding *et al.*, 2019)). Overexpression of the CBFs causes constitutive FT without the requirement for CA (Jaglo‐Ottosen *et al.*, 1998) although a major obstacle preventing CBF exploitation in crop protection has been the negative effect on growth due to their promotion of DELLA activity (Achard *et al.*, 2008). For this reason, it is pertinent to search for novel routes to FT that could be used to engineer tolerant plants without such severe growth penalties.

A forward genetic screen for Arabidopsis mutants that were susceptible to freezing even after CA identified a number of *sensitive‐to‐freezing* (*sfr*) mutants (Warren *et al.*, 1996) and several *SFR* genes have been cloned and their contribution to FT elucidated. SFR2 acts via chloroplast membrane lipid remodelling (Moellering *et al.*, 2010), SFR3 affects cuticle wax deposition (Amid *et al.*, 2012) and *SFR6* encodes the MED16 subunit of the Mediator transcriptional coactivator complex (Knight *et al.*, 2009; Hemsley *et al.*, 2014) and controls the expression of CBF‐regulated genes.

In this study, we present the mapping and cloning of a novel FT gene, *SENSITIVE‐TO‐FREEZING8 (SFR8)*. We demonstrate that reduced pectin fucosylation and crosslinking in the CW of *sfr8* mutants is associated with a compromised ability to tolerate freezing temperatures, identifying the CW as a target for the genetic control of FT.

## Materials and Methods

### Plant materials

*bor1‐3* (SALK_37312), *bor2‐1* (SALK_56473) and a *bor1‐3bor2‐1* double mutant were a kind gift from Kyoko Miwa (Hokkaido University). *mur2, mur1‐1* and *mur1‐2* (N6243, N6244 and N8565) were obtained from the Nottingham Arabidopsis Stock Centre (NASC).

### Sequencing of the *sfr8* mutant genome and analysis of polymorphisms

Illumina‐based whole‐genome sequencing of *sfr8* genomic DNA was carried out at The Genome Analysis Centre (now the Earlham Institute, Norwich, UK). The raw data were analysed using the open source platform Galaxy (https://main.g2.bx.psu.edu/) and mapped to the TAIR10 reference genome (Lamesch *et al.*, 2012) with the mapping tool Bowtie (https://main.g2.bx.psu.edu/) using the default settings. The output was read using the Integrative Genomics Viewer (IGV) software (http://www.broadinstitute.org/software/igv/). The threshold was set manually to 0.7 in order to detect mutations that appear in 70% of reads or more. The previously defined mapping interval was scanned manually for bases that differed from the reference genome sequence. After this analysis, four SNPs remained candidates for the *sfr8* mutation (Supporting Information Table S1).

### Production of the complementation lines

The *MUR1* coding sequence was amplified from Col‐0 wild‐type cDNA using the primers 5′‐CACCATGGCGTCAGAGAACAACG‐3′ and 5′‐TCAAGGTTGCTGCTTAGCATC‐3′ and cloned into the Gateway entry vector pENTR‐D‐TOPO before transfer to the destination vector pK7WG2 (Karimi *et al.*, 2002) to allow expression in plants under the control of the 35S CaMV promoter. *sfr8* mutant plants were transformed with the construct using the floral dip method (Clough & Bent, 1998) and kanamycin‐resistant transformants selected.

### Plant growth and visual freezing assay

Seeds were sown on MS agar medium as described previously (Hemsley *et al.*, 2014). After 8–10 d seedlings were transferred to 44‐mm peat plugs (LBS Horticulture, Colne, UK) and grown in short days (20°C; 12 h : 12 h, light : dark; 150‐200 µE m^−2^ s^−1^ light) for a further 4 wk before transfer to acclimating conditions (5°C; 10 h : 14 h, light : dark; 150 µE m^−2^ s^−1^ light), if used, for 2 wk. Plants were then transferred to a freezing chamber set to −8.5°C for 24 h in darkness. Plants were removed and allowed to thaw at 4°C for 8 h. For boron (B) supplementation experiments, plants were grown on half‐strength MS medium to reduce levels of available B then transferred to peat plugs at 8–10 d old and grown in short days as before. Plants were watered once a wk with deionised water with or without 20 mg l^−1^ boric acid (BA) supplementation. Further deionised water was supplied as necessary. Plants were transferred to cold acclimating conditions at 5 wk with the same supplementation regime for 2 wk before freeze testing.

### Electrolyte leakage assays

Plants grown and cold‐acclimated (where applicable) as described above were subjected to an electrolyte leakage (EL) assay as we have described previously (Hemsley *et al.*, 2014) but modified to use leaf discs, except in the experiments using *bor* mutants, which had smaller leaves. Six replicate samples per genotype per temperature tested were prepared, each consisting of three 8‐mm‐diameter leaf discs taken from the same plant using a cork borer.

### Inhibition of fucosylation with 2f‐fucose

Peracetylated 2‐fluoro‐2‐deoxy‐l‐fucose (2f‐fucose, Merck Millipore, Nottingham, UK) was dissolved in DMSO to give a stock solution of 10 mM. Wild‐type Arabidopsis (Col‐0) seedlings were grown on half‐strength MS medium supplemented with 2f‐fucose at 2.5 µM, 10 µM or 25 µM or 0.25% (v/v) DMSO (control, corresponding to the highest concentration of DMSO present in the 2f‐fucose treatments). Seedlings were grown for 14 d before being assessed for freezing damage using the EL assay. Approximately 10 mg of seedlings was used for each of the replicate samples.

### Cell‐wall sugar analysis

Plants were grown on full‐strength MS agar medium for 14 d as described above and cell wall sugars extracted following standard procedures (York *et al.*, 1986), outlined in detail below. Approximately 100 mg of tissue was harvested and ground in 70% (v/v) ethanol at 80°C to remove the alcohol‐soluble fraction, and then dried overnight in a vacuum dryer. Samples were then rehydrated in 500 µl water in a sonicating water bath before the addition of 100 µg inositol as internal standard and samples incubated in 2 M trifluoroacetic acid for 2 h at 110°C. After this, 800 µl of supernatant was transferred to glass vials and dried under N_2_ at 40°C. Once dry, 400 µl of 1 M hydrochloric acid in methanol was added and samples incubated at 80°C overnight. Samples were then dried under N_2_ at 40°C until completely dry then 400 µl of 1‐(trimethylsilyl)imidazole/pyridine 1 : 4 (v/v) (Sigma, Poole, Dorset, UK) mixture added and incubated at 80°C. After 30 min, samples were dried under N_2_ at 40°C; the residue was suspended in 1 ml hexane and vortexed vigorously. Samples were centrifuged and transferred to clean glass vials to remove the salt, before partitioning with an equal volume of water to hexane. After vortexing vigorously, an upper and lower phase was generated, with the upper hexane phase being transferred to GC‐MS vials for analysis. The GC‐MS analyses were performed using a single‐quadrupole Shimadzu QP‐2010‐Plus system fitted with a Restek Rxi‐5Sil column (30 m, 0.25 mm ID). Samples were introduced by split injection and the carrier gas was helium. The injector temperature was 250°C and the initial oven temperature was 140°C, increasing at 2°C min^−1^ to 180°C and held at this temperature for 5 min before increasing to 275°C at 10°C min^−1^, held for 10 min. Seven monosaccharides (arabinose, fucose, galactose, glucose, mannose, xylose and inositol obtained from Supelco and Sigma‐Aldrich) were used as reference standards as described previously (Lobine *et al.*, 2018). Quantification was carried out using inositol as the internal standard.

### RGII analysis by gel electrophoresis

Leaves were harvested from 5‐wk‐old plants, ground in liquid nitrogen and *c.* 50 mg of the ground tissue used to prepare alcohol‐insoluble residue (AIR). Tissue was washed twice in 96% ethanol (v/v) at room temperature, followed by a second incubation in fresh ethanol for 16 h at 37°C. Samples were then incubated in 40 ml of 1 M Na_2_CO_3_ (pH 11.5) for 16 h at 4°C after which samples were acidified with a slight excess of acetic acid, washed in 500 µL of acetone and dried overnight. The AIR (*c*. 5 mg) was digested in 1 ml of 2 U ml^−1^ endopolygalacturonase (EPG; Megazyme, Irishtown, County Wicklow, Ireland) suspended in pyridine/acetic acid/water (1 : 1 : 98) with 0.5% chlorobutanol for 16 h at room temperature and the pectin digest separated using polyacrylamide gel electrophoresis as described in Chormova *et al.* (2014). Briefly, 12 µl of sample was mixed with 3 µl of buffer (0.63 M Tris‐HCl, 0.25% (w/v) bromophenol blue, 50% (v/v) glycerol, pH 8.8) and electrophoresed through a 26.4% polyacrylamide gel (buffer 50 mM Tris‐HCl, 38 mM glycine, pH 9) for 75 min at 200 V. The gel was then fixed in ethanol/acetic acid/water (4 : 1 : 5) for 30 min then washed with water three times for 1 min followed by treatment with 400 µM sodium thiosulphate (1 min), water (1 min, three times), 6 mM silver nitrate in 10 mM formaldehyde (20 min), water (20 s, twice) and 0.28 M Na_2_CO_3_ containing 8 µM sodium thiosulphate and 64 mM formaldehyde for 2–10 min. Colour development was stopped by addition of 0.33 M Tris base in 2% (v/v) acetic acid. RGII monomer and dimer standards were included for comparison.

### Statistical analyses

For EL experiments, percentage electrolyte leakage values from two or three biological replicate experiments were arcsine‐transformed. A linear mixed effects model (Kuznetsova *et al.*, 2016) was computed using R software (R Core Team, 2016), with genotype and any treatment (i.e. BA supplementation) specified as fixed terms, and experiment specified as a random effect. For the BA supplementation experiments, results were analysed by a two‐way ANOVA at each temperature point with an interaction term specified between genotype and BA. For other EL experiments, a one‐way ANOVA was carried out to determine the effect of genotype on the level of EL. Significant differences in leakage between genotypes and/or treatments was assessed using a least‐squares means comparison (Lenth, 2016) at each temperature datapoint. A one‐way ANOVA and least‐squares means comparison was also carried out to assess the significance of cell‐wall fucose levels in different genotypes, with experiment specified as a random effect.

## Results

### Identification of *sensitive‐to‐freezing8* as an allele of *mur1*

**Figure 1:**
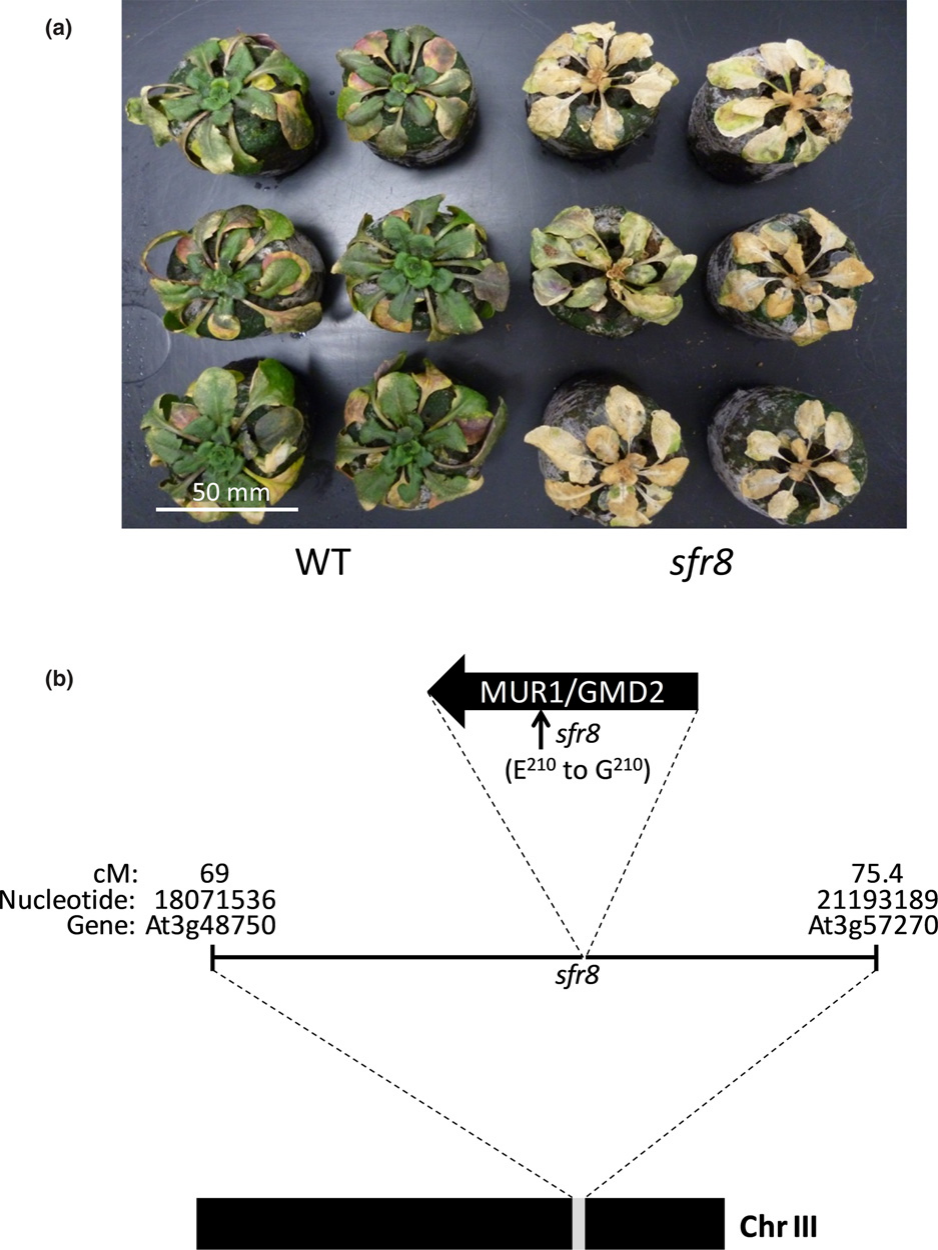
Fine mapping of the *sensitive‐to‐freezing8* (*sfr8*) mutation. (a) Cold‐acclimated Col‐0 wild‐type (WT) *Arabidopsis thaliana* and *sfr8* mutant plants 1 wk after a 24‐h freezing treatment at −8.5°C. Plants were grown for 5 wk before acclimating at 5°C for 2 wk. (b) Diagrammatic representation of the interval determined on chromosome III as containing the *sfr8* mutation.

A screen for ethyl methanesulphonate (EMS)‐induced Arabidopsis mutants that were freezing‐sensitive even after cold acclimation (CA) was carried out previously, identifying *sensitive‐to‐freezing* mutations 1‐7 (*sfr1‐7*) (Warren *et al.*, 1996). *sfr8* and *sfr9* were subsequently isolated (Thorlby *et al.*, 1999). 1 wk after a 24‐h freezing treatment at −8.5°C, cold‐acclimated wild‐type (WT) plants showed signs of recovery and the majority of leaf tissue was green whilst *sfr8* mutant plants exhibited almost complete chlorosis and failed to regrow subsequently (Fig. 1a). The *sfr8* mutation had no obvious effect on the expression of the *CBF* genes or the two CBF‐controlled *COR* genes *KIN2* and *GOLS3* (Fig. S1), indicating that SFR8 was unlikely to control freezing tolerance (FT) by acting upstream of the CBFs. *SFR8* was previously mapped to chromosome 3 (Thorlby *et al.*, 1999) between markers CDC2a (69 cM) and BGL1 (75.4 cM); equating to the interval between the genes At3g48750 and At3g57270 (Fig. 1b). We took an Illumina whole‐genome sequencing approach to identifying SNPs within this interval and we mapped these to the TAIR10 Arabidopsis reference genome (Lamesch *et al.*, 2012). SNPs appearing in <70% of reads were disregarded. After quality control, four SNPs were identified in the interval as having the potential to be the *sfr8* mutation (Table S1). Sanger sequencing of the regions containing these putative SNPS confirmed all four of them to be present in *sfr8* genomic DNA and homozygous. One of these (Chr3:18684521) did not fall within an annotated gene and so was not pursued further; a second SNP, at Chr3:20966136, fell within an intron and was considered unlikely to be the cause of the *sfr8* phenotype (Table S1). The remaining two SNPs were in two genes: At3g50910 and At3g51160. We obtained homozygous insertional mutants for these genes from NASC. Two homozygous insertions into At3g50910 were confirmed as exhibiting reduced transcript levels: SALK_074693C and SALK_132810C (Fig. S2). These were tested for FT but showed no difference to WT plants, indicating that the *sfr8* mutation was not in At3g50910 (Fig. S2).

**Figure 2:**
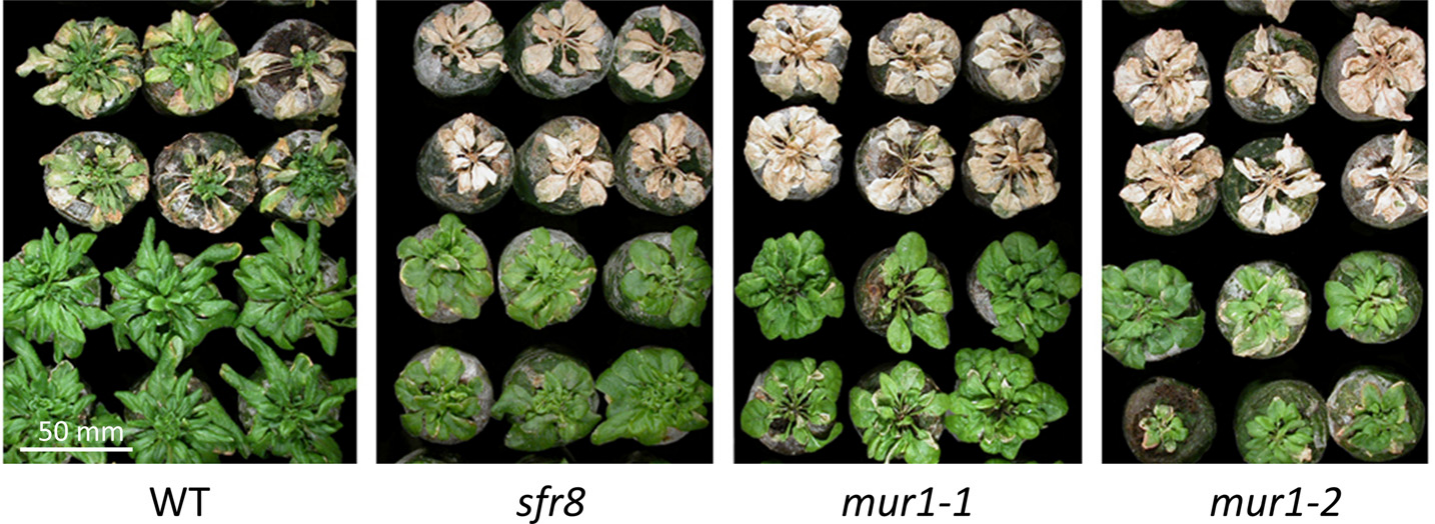
*sfr8* and *mur1* mutants are both freezing‐sensitive. Cold‐acclimated Col‐0 wild‐type (WT) *Arabidopsis thaliana* and *sfr8* mutant plants alongside *mur1‐1* and *mur1‐2* mutants 1 wk after a 24‐h freezing treatment at −8.5°C (upper two rows) or without freezing (lower two rows). Plants were grown for 5 wk before acclimating at 5°C for 2 wk.

For At3g51160 (*MUR1*), we were unable to isolate viable homozygous mutants from any of three insertion lines (SALK_027379, SALK_027387 and SALK_027279); therefore, we obtained two EMS lines, *mur1‐1* and *mur1‐2* (Reiter *et al.*, 1993) (Fig. S3). *mur1‐1* and *mur1‐2,* like *sfr8*, were more freezing‐sensitive than WT after CA (Fig. 2). We also observed that all three mutants displayed a similar rounded leaf shape when compared to Col‐0 WT (Fig. 2). The *sfr8*, *mur1‐1* and *mur1‐2* mutations all result in amino acid substitutions to the MUR1 protein; *sfr8* is identical to the previously described *mur1‐4* (Fig. S3; Bonin *et al.*, 1997). These observations strongly indicated that *sfr8* is a mutant in the *MUR1* gene and, therefore, MUR1 confers FT.

**Figure 3:**
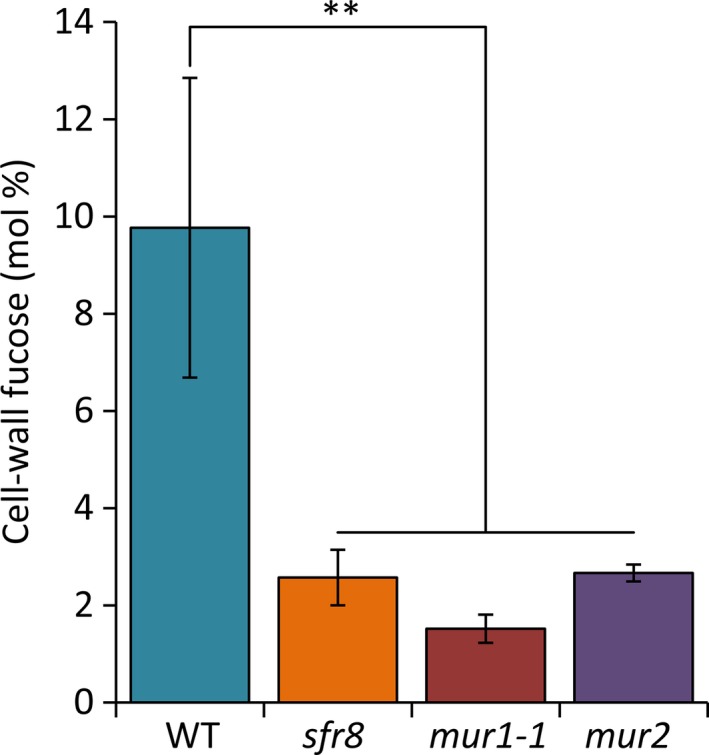
Cell‐wall fucose levels are reduced in *sfr8, mur1‐1* and *mur2* mutants. Col‐0 wild‐type (WT) *Arabidopsis thaliana*, *sfr8*, *mur1‐1* and *mur2* plants were grown on MS agar for 14 d after which samples of alcohol insoluble residue (AIR) were prepared and cell‐wall fucose content (mol%) analysed by GC‐MS. Data presented are the mean values from two independent biological replicates and were analysed using a least‐squares means comparison (**, *P* < 0.01). Error bars represent ± 1 SE.

*MUR1* was originally identified in a forward genetic screen for cell wall (CW) mutants, in which mutants were selected on the basis of altered CW polysaccharide composition, with *mur1* exhibiting vastly reduced levels of shoot CW fucose (Reiter *et al.*, 1993). This is because *MUR1* encodes an isoform of GDP‐d‐mannose‐4,6‐dehydratase (GMD2), the enzyme catalysing the first step in the *de novo* synthesis of activated fucose, GDP‐l‐fucose (Bonin *et al.*, 1997). Using a thin‐layer chromatography assay developed by Bonin *et al.* (1997), we showed that, as expected, extracts from *sfr8* mutant plants were unable to convert GDP‐d‐mannose substrate into GDP‐l‐fucose, unlike WT plants, indicating that the mutation in *MUR1* found in *sfr8* results in the production of non‐functional GDP‐d‐mannose‐4,6‐dehydratase (Fig. S4)*.* Two other *sfr* mutants, including *sfr4*, which is deficient in sucrose accumulation (Uemura *et al.*, 2003), showed WT levels of conversion (Fig. S4). As a result of reduced fucose synthesis *mur1* mutants show reduced incorporation of fucose into CW polysaccharides (Reiter *et al.*, 1993) and glycoproteins (Rayon *et al.*, 1999). We found CW fucose incorporation in *sfr8* mutants was reduced to levels similar to those seen in *mur1‐1* (Fig. 3). A one‐way ANOVA followed by a least‐squares means comparison showed that fucose levels were significantly decreased compared to WT plants (*P* < 0.01).

**Figure 4:**
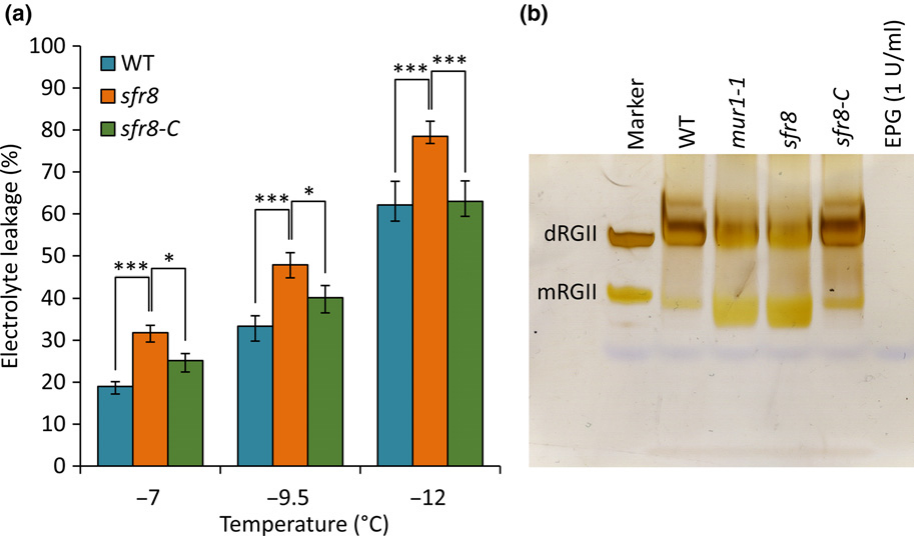
*sfr8* can be complemented by *MUR1.* (a) Electrolyte leakage values from Col‐0 wild‐type (WT) *Arabidopsis thaliana*, *sfr8* and *sfr8‐C* (complemented line). Plants were grown for 5 wk before acclimating at 5°C for 2 wk. Values represent percentage loss of electrolytes from leaf discs when exposed to temperatures of −7, −9.5 and −12°C. Each data point represents the average of three separate biological replicate experiments. Each experiment used six replicate tubes per genotype per temperature, with three leaf discs per tube. Arcsine‐transformed percentage leakage data were analysed by a least‐squares means comparison at each temperature point (*, *P* < 0.05; ***, *P* < 0.001). Error bars represent ± 1 SE of arcsine‐transformed data. (b) The proportion of RGII in the dimerized form is greater in WT *Arabidopsis thaliana* and *sfr8*‐C plants than it is in *mur1‐1* or *sfr8* mutants, which show higher proportions of the monomer form. Col‐0 wild‐type (WT), *mur1‐1*, *sfr8* and *sfr8‐*C plants were grown at 20°C for 5 wk. Samples of alcohol insoluble residue (AIR) were produced from leaves of plants and digested with endopolygalacturonase (EPG), and products analysed by polyacrylamide gel electrophoresis. The first lane on the gel shows RGII monomer (mRGII) and dimer (dRGII) standards (0.8 µg of each were loaded). mRGII and dRGII products were stained with silver nitrate.

To confirm finally that *SFR8* and *MUR1* were allelic, we created complemented lines by transforming *sfr8* mutant plants with a vector containing the *MUR1* coding sequence under the control of the 35S CaMV promoter. We identified 3 independent complemented lines (lines 1, 8 and 14) with FT restored to WT levels (Fig. 4, Fig. S5a). Line 14 was selected for further study (referred to hereafter as *sfr8*‐*C*). We confirmed that the low levels of CW fucose observed in *sfr8* mutants were restored to WT levels in *sfr8*‐*C* plants (Fig. S6). RGII dimerization, which is dependent on fucosylation of RGII domains, was also restored in *sfr8*‐*C* (Fig. 4b). Levels of FT after CA were assessed in the mutant and line *sfr8*‐*C* using a quantitative electrolyte leakage (EL) assay to measure the degree of cellular damage after freezing. A one‐way ANOVA showed a significant effect of genotype on EL for all three temperatures tested over three biological replicate experiments (***, *P* < 0.001; *, *P* < 0.05; Fig. 4a). *sfr8* mutants showed greater leakage than WT plants at the three freezing temperatures tested, consistent with greater sensitivity to freezing. However, the complemented line, *sfr8‐C*, showed significantly lower levels of leakage than *sfr8* mutants, similar to those of WT plants, indicating that the *MUR1* gene could complement the freezing‐sensitive phenotype of *sfr8.* Consistent with these quantitative measures of damage, the WT and complemented line showed less visible damage after freezing than did the *sfr8* mutant (Fig. S5b). These data allowed us to confirm *SFR8* as *MUR1* and to show that *MUR1* plays a role in FT.

### A fucosylation event is necessary for full freezing tolerance

Having demonstrated genetic linkage between the *MUR1*/*SFR8* gene, CW fucose content, RGII dimerization and FT we used an EL assay to test for the ability of supplementary fucose to restore FT in *mur1* or *sfr8* mutants. FT was restored in *mur1‐1* and *sfr8* mutants that had been sprayed with supplementary fucose (Fig. S7), confirming that low levels of cellular fucose are linked to freezing sensitivity. Application of fucose to WT plants did not alter EL, indicating that at the levels applied, fucose supplementation had no significant effect *per se* on WT tolerance (for instance, as a compatible solute).

**Figure 5:**
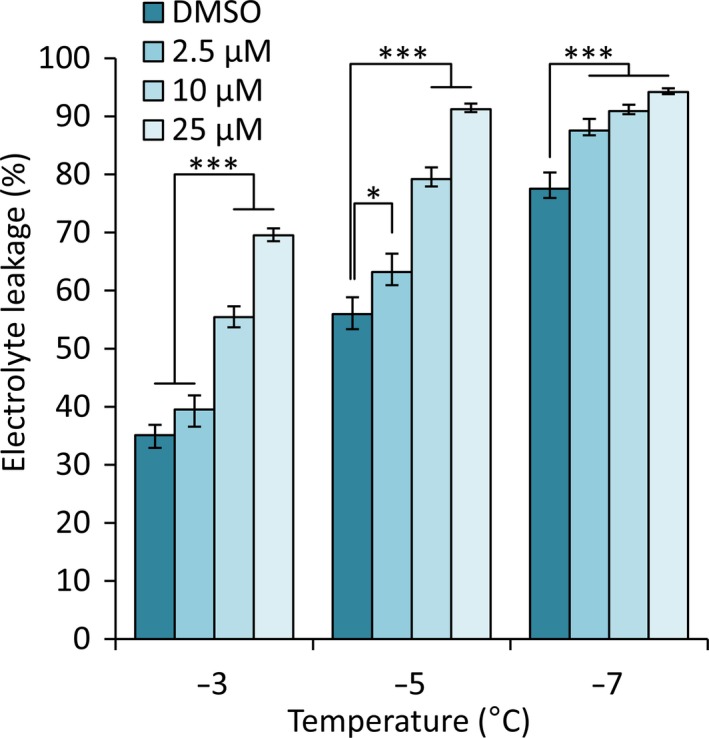
Freezing tolerance is reduced by 2f‐fucose, a fucose synthesis inhibitor, in a concentration‐dependent manner. Electrolyte leakage values from Col‐0 wild‐type (WT) *Arabidopsis thaliana* plants treated with DMSO and three different concentrations of 2f‐fucose. Plants were grown on MS agar containing DMSO, 2.5 µM, 10 µM or 25 µM of 2f‐fucose for 2 wk. Values represent percentage loss of electrolytes from seedlings when exposed to temperatures of −3, −5 and −7°C. Each data point represents the average of three separate biological replicate experiments. Each experiment used six replicate tubes per treatment per temperature, with *c.* 10 mg seedlings per tube. Arcsine‐transformed percentage leakage data were analysed by a least‐squares means comparison at each temperature point (*, *P* < 0.05; ***, *P* < 0.001). Error bars represent ± 1 SE of arcsine‐transformed data.

Our data indicated that either the level of free fucose levels or fucosylation of other molecules was important for maintaining WT levels of FT. To test whether fucosylation contributes to FT we assessed the effect of an inhibitor of fucosylation, 2‐fluoro‐2‐l‐fucose (2f‐fucose) (Dumont *et al.*, 2015; Villalobos *et al.*, 2015) on WT plants. Non‐acclimated seedlings grown on agar supplemented with the inhibitor were subjected to EL analysis to assess their FT. EL analysis of non‐acclimated plants is, from necessity performed at higher temperatures than those used for acclimated plants, as they are less freezing tolerant (Gilmour *et al.*, 2000). Similarly, testing seedlings rather than mature rosette plants necessitates the use of less severe temperatures (Xin & Browse, 1998). A one‐way ANOVA showed a significant effect of 2f‐fucose treatment at all temperatures tested (***, *P* < 0.001, *, *P* < 0.05, Fig. 5). The two higher concentrations of 10 and 25 µM were effective in increasing the damage to WT Arabidopsis frozen at −3 and −5°C, whilst all three concentrations were effective at −7°C (Fig. 5). These data indicate that fucosylation contributes to FT but they do not allow identification of the specific fucosylation events that are required.

A number of CW components undergo fucosylation in WT plants, including arabinogalactan proteins (AGPs), xyloglucans (XyG) and pectins (Nakamura *et al.*, 2001; Perrin *et al.*, 2003; Wu *et al.*, 2010). In order to ascertain whether the reduced FT we observed in *sfr8* was linked to a general lack of fucosylation in the CW or to the fucosylation of one particular component, we investigated the effect of the *mur2* mutation on FT. *mur2* mutants have a mutation in the *FUT1* gene, which encodes a XyG‐specific fucosyltransferase that fucosylates the hemicellulosic CW component XyG (Vanzin *et al.*, 2002). Whilst *mur2* mutants showed reduced fucose incorporation into the CW fraction (Fig. 3) they exhibited minimal differences in freezing sensitivity (Fig. S5b, S8). This indicates that it is unlikely that fucosylation of XyGs plays a role in plant FT, but does not eliminate the possibility that either or both AGP and pectin fucosylation might do.

### Borate bridging of the pectic domain rhamnogalacturonan II is associated with freezing tolerance

Plant CW pectins comprise three main domains: homogalacturonans (HG) and rhamnogalacturonans I and II (RGI and RGII; Harholt *et al.*, 2010). Arabidopsis RGII domains have six side chains (side chains A to F; (Ndeh *et al.*, 2017)), two of which are usually fucosylated in WT plants (O'Neill *et al.*, 2001). *mur1* mutants lack fucosylation of side chain A and exhibit reduced growth and CW mechanical strength as a result (Reiter *et al.*, 1993; O’Neill *et al.*, 2001). Fucosylation of side chain A is necessary if RGII domains are to dimerize efficiently and stably via a borate‐diester crosslink formed between the apiose residues of two RGII A chains (Kobayashi *et al.*, 1996). Borate bridging of RGII domains mainly occurs before or during secretion of the pectic polysaccharides into the wall (Chormova *et al.*, 2014), probably in association with plasma‐membrane glycolipids (Voxeur & Fry, 2014). In *mur1* mutants l‐galactose substitutes for l‐fucose in side chain A (Reuhs *et al.*, 2004) but the side chain is truncated (Pabst *et al.*, 2013; Sechet *et al.*, 2018) and dimerization is consequently reduced (O'Neill *et al.*, 2001; Voxeur *et al.*, 2017; Sechet *et al.*, 2018).

**Figure 6:**
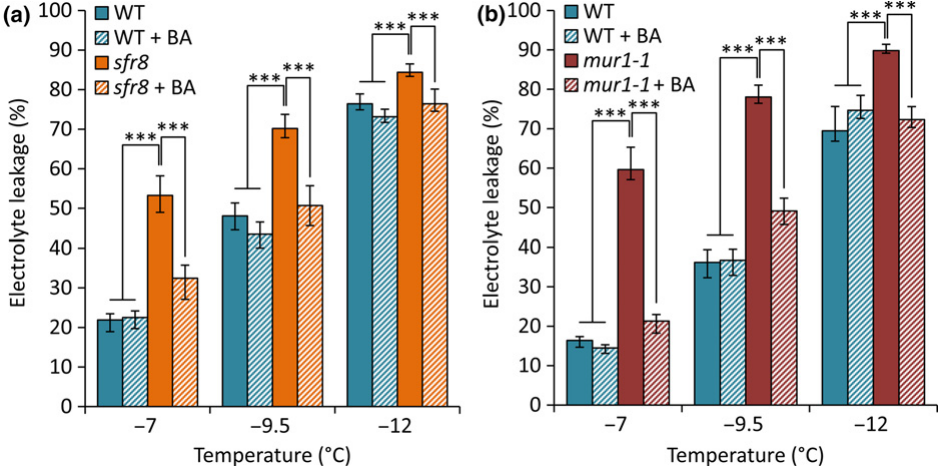
Addition of boric acid restores freezing tolerance in *sfr8* and *mur1‐1* mutant plants. Electrolyte leakage values from Col‐0 wild‐type (WT) *Arabidopsis thaliana*, (a) *sfr8* and (b) *mur1‐1* plants with and without boric acid (BA) supplementation. Plants were grown for 5 wk before acclimating at 5°C for 2 wk. Values represent percentage loss of electrolytes from leaf discs when exposed to temperatures of −7, −9.5 and −12°C. Each data point represents the average of three separate biological replicate experiments. Each experiment used six replicate tubes per genotype/treatment per temperature, with three leaf discs per tube. Arcsine‐transformed percentage leakage data were analysed by a least‐squares means comparison at each temperature point (***, *P* < 0.001). Error bars represent ± 1 SE of arcsine‐transformed data.

Restoration of *mur1* phenotypes by boron (B) supplementation has been recognised as confirmation that a phenotype is associated specifically with reduced borate bridging of RGII due to the lack of fucose in side chain A, rather than attributable to a reduction in other fucosylation events (O'Neill *et al.*, 2001; Ryden *et al.*, 2003; Voxeur *et al.*, 2017; Feng *et al.*, 2018; Sechet *et al.*, 2018). RGII crosslinking, growth and the tensile strength breaking force of *mur1* mutant inflorescences were all restored to levels approaching those of WT plants in *mur1* mutants by supplementation with additional boric acid (BA) (O'Neill *et al.*, 2001; Ryden *et al.*, 2003; Sechet *et al.*, 2018). To test whether FT could be restored by BA, we grew WT, *mur1‐1* and *sfr8* plants with or without BA supplementation and cold‐acclimated them. The visible phenotype associated with mutation of the *MUR1* gene was restored in mutant plants by BA supplementation (Fig. S9); in the absence of supplementation mutants exhibited the reduced petiole length and rounded, non‐serrated leaves typical of *mur1* (O'Neill *et al.*, 2001; Goncalves *et al.*, 2017)*.* Whilst non‐supplemented *sfr8* and *mur1‐1* mutants showed the expected freezing‐sensitive phenotype and greater levels of EL than WT, *sfr8* and *mur1‐1* mutants supplemented with BA both showed reduced levels of damage similar to WT plants. A two‐way ANOVA showed a significant effect of genotype on EL (*P* < 0.001) and a significant interaction of genotype with BA (*P* < 0.05) for all temperatures tested, highlighting the fact that BA reduced the EL of both mutants, but had no effect on WT plants. A least‐squares means comparison showed that *sfr8* and *mur1‐1* leakage was significantly greater than leakage from WT and BA‐supplemented WT plants or BA‐supplemented mutant plants (*P* < 0.001) (Fig. 6a,b). These data suggested that RGII dimerization is required for full WT levels of FT.

### Cell‐wall fucosylation, and most likely RGII crosslinking, contribute to basal freezing tolerance

**Figure 7:**
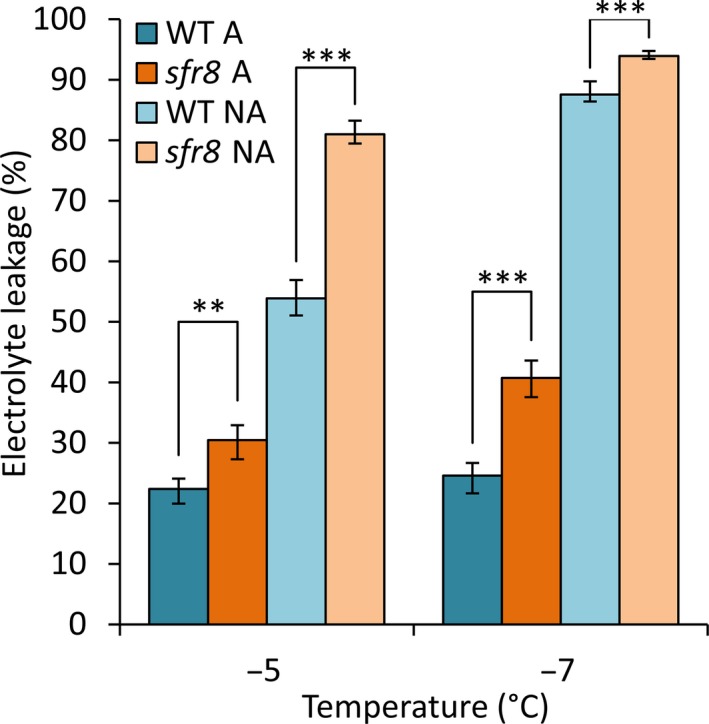
*sfr8* mutants are more susceptible to freezing than WT with and without cold acclimation. Electrolyte leakage values from Col‐0 wild‐type (WT) *Arabidopsis thaliana* and *sfr8*. Non‐acclimated (NA) plants were grown for 5 wk, cold acclimated (A) plants were grown for 5 wk and then acclimated at 5°C for 2 wk. Values represent percentage loss of electrolytes from leaf discs when exposed to temperatures of −5 and −7°C. Each data point represents the average of two separate biological replicate experiments. Each experiment used six replicate tubes per genotype per temperature, with three leaf discs per tube. Arcsine‐transformed percentage leakage data were analysed by a least‐squares means comparison at each temperature point (**, *P* < 0.01; ***, *P* < 0.001). Error bars represent ± 1 SE of arcsine‐transformed data.

Our data raised the possibility that *MUR1*‐mediated RGII dimerization might be part of the WT CA process that leads to improved FT. Using the Genevestigator tool (genevestigator.com/gv/) (Zimmermann *et al.*, 2004) we found that *MUR1* was not upregulated by overexpression of the CA‐specific transcription factors CBF2 (Vogel *et al.*, 2005) or CBF3 (Chan *et al.*, 2012) (Table S2). Neither was *MUR1* misregulated in the *cbfs* triple mutant (Jia *et al.*, 2016). Consistent with this, a search for transcription factor binding sites within the *MUR1* promoter using the AGRIS promoter database (agris‐knowledgebase.org/cite.html) (Yilmaz *et al.*, 2011) revealed no CBF binding motifs. This indicated that *MUR1* is not a target of the CBF transcription factors but did not eliminate the possibility that it could be upregulated in response to CA independently of the CBFs. However, we found that *MUR1* was not induced by exposure to 5°C for 1, 3, 6 or 12 h or 1, 4 or 7 d (Fig. S10a), unlike the cold‐inducible gene *KIN2*. This was consistent with published transcriptomic data that shows no significant increase in *MUR1* transcript levels in plants transferred to CA conditions (Fig. S10b; Table S3; (Calixto *et al.*, 2018)) and with reports that fucose levels do not increase during CA in Arabidopsis (Cook *et al.*, 2004; Takahashi *et al.*, 2019) or *Pisum sativum* (Baldwin *et al.*, 2014). Therefore, we hypothesised that *MUR1* is unlikely to contribute to FT by increasing fucose levels during CA and is more likely to influence basal FT. We confirmed that *sfr8* was more *sensitive‐to‐freezing* at −5 or −7°C than WT even when not acclimated (*P* < 0.001) and that its tolerance of these temperatures did improve significantly after CA (*P* < 0.001) but failed to reach WT levels (Fig. 7). Consistent with our findings, non‐acclimated *sfr8* were also more susceptible to freezing than WT at less severe freezing temperatures (−2 and −4°C) (Fig. S11).

**Figure 8:**
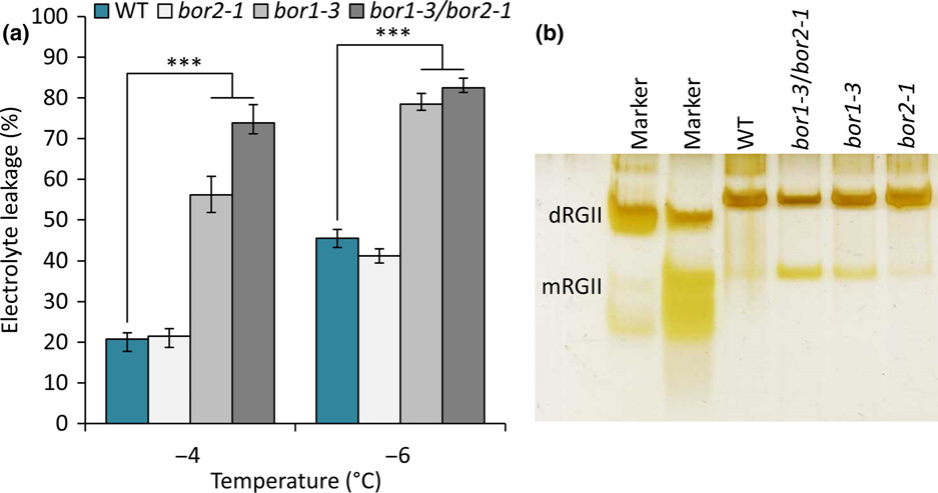
Boron transporter mutants are susceptible to freezing. (a) Electrolyte leakage values from Col‐0 wild‐type (WT) *Arabidopsis thaliana*, *bor2‐1*, *bor1‐3* and *bor1‐3bor2‐1* plants. Plants were grown for 5 wk under short day conditions and boron‐limiting conditions and not acclimated. Values represent percentage loss of electrolytes from leaves when exposed to temperatures of −4 and −6°C. Because *bor* mutants are smaller than wild‐type plants, experiments were conducted using size‐matched leaves and repeated using age‐matched leaves and the same results were observed. Each data point represents the average of three separate biological replicate experiments. Each experiment used six replicate tubes per genotype per temperature, with three leaves per tube. Arcsine‐transformed percentage leakage data were analysed by a least‐squares means comparison at each temperature point (***, *P* < 0.001). Error bars represent ± 1 SE calculated from arcsine‐transformed data. (b) The proportion of RGII in the dimerized form is greater in WT and *bor2‐1* plants than it is in the *bor1‐3* or *bor1‐3bor2‐1* mutants, which show higher proportions of the monomerised form. Samples of alcohol insoluble residue (AIR) were produced from leaves of plants and digested with endopolygalacturonase (EPG), and products analysed by polyacrylamide gel electrophoresis. The first lanes on the gel show RGII monomer (mRGII) and dimer (dRGII). mRGII and dRGII products were stained with silver nitrate.

Our data indicated that fucose‐dependent borate‐dimerization of RGII plays a role in FT irrespective of CA. To add further support to this conclusion we tested other mutants defective in borate dimerization of RGII for damage following freezing without prior CA. BOR1 and BOR2 are plasma membrane B transporters and mutants in either *BOR1* or *BOR2* require supplementary BA to maintain normal wild‐type growth and CW structure (Miwa *et al.*, 2013). After growth under B‐limiting conditions *bor1‐3* and a double mutant *bor2‐1bor1‐3* both showed reduced RGII dimerization, as evidenced by a higher monomer : dimer ratio (Fig. 8b). Levels of EL were significantly higher in freeze‐thawed leaves of these mutants (*P* < 0.001) than in leaves of WT plants subjected to the same freezing temperatures (Fig. 8a). Together, these data strongly indicate that the lack of fucose in *sfr8* plants results in freezing sensitivity specifically as a consequence of reduced RGII dimerization.

## Discussion

Research into the genetic control of plant freezing tolerance (FT) has, to date, focussed largely on the damage to cellular membranes and the cytoplasmic dehydration that occur during freezing stress. Here we describe the identification of a novel FT gene, *SFR8*, through fine mapping and genome sequencing of a *sensitive‐to‐freezing* EMS mutant and we demonstrate that SFR8 acts through modifying the composition and function of the plant primary cell wall (CW).

Other studies have implicated the CW in regulating FT. For example, reduced biosynthesis of lignin, a component of the secondary CW, results in greater FT (Ji *et al.*, 2015). *SFR8* (At3g51160/*MUR1*) was originally identified in a screen for mutants with altered CW sugar composition (Reiter *et al.*, 1993). Levels of fucose in the shoot of *mur1* mutants were barely detectable and *mur1‐1* mutants exhibited reduced mechanical strength and growth (Reiter *et al.*, 1993; Zablackis *et al.*, 1996). MUR1 was subsequently identified as GMD2, an isoform of the first enzyme in the biosynthetic pathway for GDP‐l‐fucose (Bonin *et al.*, 1997). We show that CW‐fucose incorporation is reduced in *sfr8* mutants similarly to *mur1‐1* (Fig. 3). We show that *SFR8* is allelic with *MUR1; mur1‐1*, *mur1‐2* and *sfr8* representing three different SNPs in At3g51160 that each cause a different amino acid substitution in the MUR1 protein (Fig. 2, Figs S3, S5b). *mur1* and *sfr8* plants exhibited a similar physical appearance to one another, *sfr8* exhibiting the cup‐shaped cotyledons and rounded leaves that have been previously reported for *mur1* mutants and associated with the effects of reduced fucosylation (O'Neill *et al.*, 2001; Goncalves *et al.*, 2017) (Fig. 2); however, they were not severely dwarfed as reported previously (O'Neill *et al.*, 2001). We consider this likely to be due to the fact we grew our plants under short day conditions (typical for CA experiments), and our growth regime may have been less B‐limiting than those used previously. FT was impaired in fucose‐deficient *mur1*/*sfr8* mutants but this could be restored by spraying with fucose (Fig. S7). By using 2f‐fucose, a competitive inhibitor of a broad spectrum of cellular fucosylation events (Dumont *et al.*, 2015; Villalobos *et al.*, 2015) we were able to mimic the effect of the *sfr8* mutation, observing increased tissue damage after freezing in WT seedlings (Fig. 5). This suggests that FT is dependent on a fucosylation event rather than on fucose itself.

Both the protein and polysaccharide components of the CW are subject to fucosylation in WT plants, with *N*‐linked glycoproteins, arabinogalactan proteins (AGPs), the hemicellulose xyloglucan (XyG) and the pectic domains RGI and RGII all being targets. Previous work has shown that in *mur1* mutants l‐fucose (l‐fuc) and 2‐*O*‐methyl l‐fucose are replaced by l‐galactose (l‐gal) and 2‐*O*‐methyl l‐galactose respectively in CW polysaccharides but that this substitution has little or no effect on the structure and function of XyGs (Zablackis *et al.*, 1996). This suggested that lack of XyG fucosylation is unlikely to be the cause of freezing sensitivity in *sfr8*. This view is supported by our observation that the *mur2* mutant, which lacks functional XyG fucosyl transferase, FUT1 (Vanzin *et al.*, 2002), showed little sensitivity to freezing despite the significant reduction in its CW fucose level (Figs S5b, S8).

In contrast with XyG, the structure of RGII is substantially altered by substitution of l‐fuc with l‐gal (Reuhs *et al.*, 2004). Pectic RGII in wild‐type plants is predominantly crosslinked (dimerized) via borate‐diester linkages between apiose sugars in side chain A of RGII monomers (Kobayashi *et al.*, 1996); dimerized RGII is associated with elasticity and mechanical strength in the CW (Ryden *et al.*, 2003). RGII domains are highly negatively charged (thus mutually repulsive) and the formation of crosslinks between them via borate bridges is strongly enhanced by cationic ‘chaperones’ e.g. extensins (Chormova & Fry, 2016). Substitution of l‐fuc with l‐gal in RGII results in a lower proportion of full‐length side chains, which reduces the opportunities for dimerization (O'Neill *et al.*, 2001; Pabst *et al.*, 2013). Whilst WT Arabidopsis RGII is *c*. 95% dimerized, only *c.* 50% of RGII was reported to be dimerized in *mur1* mutants (O'Neill *et al.*, 2001). Consistent with this, B deficiency causes similar CW abnormalities to those seen in *mur1* plants (Fleischer *et al.*, 1999). Our RGII analysis shows reduced RGII dimerization in *sfr8* and *mur1* plants (Fig. 4b) similar to the qualitative differences in RGII dimer : monomer ratio observed in a recent study (Voxeur *et al.*, 2017).

We, therefore, hypothesised that impaired FT in *sfr8* could be attributed to lack of RGII dimerization. Previous work has shown that RGII crosslinking can be restored in *mur1* mutants by the application of supplementary boric acid (BA) (O'Neill *et al.*, 2001; Sechet *et al.*, 2018), a treatment that also results in restoration of normal CW strength, cell integrity and growth (O'Neill *et al.*, 2001; Ryden *et al.*, 2003; Feng *et al.*, 2018). Fucose generated by MUR1 is necessary for proper establishment of leaf boundary domains by the CUP‐shaped cotyledon transcription factor CUC2 (Goncalves *et al.*, 2017). CUC2‐overexpressing lines show long leaves with serrated edges whereas the *mur1* mutation suppresses this and renders leaves more cup‐shaped. The effect of *mur1* on leaf shape is known not to be a consequence of reduced XG fucosylation and it has been suggested it may be the result of impaired RGII crosslinking (Goncalves *et al.*, 2018). The reversion of leaf shape we observe after BA supplementation would support this view (Fig. S9). Having established that our BA‐watering regime restored the fucose‐dependent growth defect in *mur1* mutants we compared FT in *sfr8* and *mur1‐1* with and without BA supplementation (Fig. 6). BA supplementation reduced the sensitivity of *sfr8* and *mur1‐1* to freeze damage, indicating that fucosylation‐dependent borate‐dimerization of RGII is required for full FT.

RGII crosslinking is the main cellular function that has been described for B in plants (O'Neill *et al.*, 2004; Funakawa & Miwa, 2015) with RGII described as possibly the only site of B binding in the CW. However, potential roles for B in CW‐membrane attachment and membrane structure have been proposed (Funakawa & Miwa, 2015) and membrane‐associated B‐interacting proteins isolated from plants (Wimmer *et al.*, 2009). It is conceivable that our BA treatment could have affected alternative B‐dependent aspects of cell structure and integrity and we cannot exclude the possibility that B may play other roles in FT that involve such interactions. However, whereas BA supplementation improved FT in *sfr8* plants, we observed no effect of BA supplementation on the FT of WT plants (Fig. 6). This indicates that the role of B in FT we observe intersects with a fucosylation event. N‐glycosylated proteins and AGPs lack fucosylation in *mur1* mutants (Rayon *et al.*, 1999; Tryfona *et al.*, 2012; Zhang *et al.*, 2018) so it remains a possibility that lack of CW protein fucosylation is responsible for the reduced FT of *mur1/sfr8*. However, no published evidence suggests that any phenotype caused by a defect in protein fucosylation can be restored by B supplementation and it is difficult to envisage a mechanism whereby this could occur. In fact the short‐root phenotype of *mur1*, attributed to reduced fucosylation of AGPs but not RGII, was not restored by B supplementation (van Hengel & Roberts, 2002). These published data and the effect of both fucosylation and B on FT together support our conclusion that the restoration of FT in *sfr8* by supplementary B is most logically explained by FT being dependent upon dimerization of fucosylated RGII.

In further support of this conclusion, we observed that the B transporter mutants *bor1‐3* and *bor2‐1bor1‐3*, which show reduced RGII‐borate dimerization whilst suffering no deficit in fucose synthesis, were freezing‐sensitive (Fig. 8). This strongly suggests that crosslinking of RGII monomers via borate‐diester linkages is a specific requirement for FT and that the freezing‐sensitivity of *mur1*/*sfr8* is not a consequence of any other impaired fucosylation event. *BOR1* and *BOR2* encode plasma membrane‐localised efflux‐type B transporters expressed in root cells (Takano *et al.*, 2002; Miwa *et al.*, 2013) and under B‐limiting conditions mutants show reduced B uptake (Miwa *et al.*, 2013). Whilst previous work showed that *bor2‐1* mutants had reduced RGII crosslinking in root cells whereas *bor1‐3* did not (Miwa *et al.*, 2013), we saw the opposite effect in rosette leaf tissue, *bor1‐3* having a noticeable effect on RGII monomer : dimer ratio in plants grown on low levels of B (Fig. S9b). A recent study showed that regenerative xylem formation occurred in response to reduced RGII dimerization in stems of *bor1‐3*, similar to *mur1* mutants (Voxeur *et al.*, 2017), therefore, it appears likely that BOR1 makes the greater contribution to RGII dimerization in aerial tissues. In addition to self‐dimerization, RGII has been reported as forming B‐containing complexes with plasma membrane glycosylinositol phosphorylceramides (GIPCs) in *Rosa* cell cultures and binding to AGP‐extensin in symbiotic infection threads via B‐dependent linkages (Reguera *et al.*, 2010; Voxeur & Fry, 2014). It is possible that fucosylated RGII contributes to FT via interactions like these as well as through RGII dimerization.

It is interesting to note recent work demonstrating a role for RGII dimerization in maintaining CW integrity in plants challenged with salinity stress (Feng *et al.*, 2018; Sechet *et al.*, 2018), suggesting a wider role for RGII crosslinking in the response to abiotic stress. RGII dimerization via borate‐diester linkages has a number of consequences in plant CWs: increasing wall thickness (Ishii *et al.*, 2001), elasticity and mechanical strength (Ryden *et al.*, 2003) and decreasing CW pore size (Fleischer *et al.*, 1999). Accordingly, restricting B supply rapidly reduces the elasticity of CWs (Findeklee & Goldbach, 1996) and renders tissues brittle (Blevins & Lukaszewski, 1998). Our data suggest that one or more of these properties may be important in determining plant FT. During CA, CW strength and thickness increase whilst pore size decreases (Rajashekar & Lafta, 1996; Kubacka‐Zebalska & Kacperska, 1999; Stefanowska *et al.*, 1999; Arias *et al.*, 2015), further supporting the suggestion that these CW properties are important for FT.

As the cell’s barrier against the external environment, the CW is the site of extracellular ice nucleation (Wisniewski *et al.*, 1997; Pearce, 2001). It has in the past been suggested that the qualitative nature of the CW may determine how easily, and where, ice can nucleate, affecting the level of freezing damage (Burke *et al.*, 1976; McCully *et al.*, 2004). Smaller pore sizes have been shown to restrict the propagation of ice within plants (Ashworth & Abeles, 1984) and CW rigidity has been associated with reduced levels of freeze‐induced dehydration (Rajashekar & Burke, 1996). Increased CW rigidity may also help the cell deal with the mechanical strain imposed by extracellular ice formation (Smallwood & Bowles, 2002). Pectin crosslinking (albeit via calcium bridges) was proposed to play a role in preventing ice nucleation back in 1991 when it was suggested it might exert this effect through modifying the CW’s permeability to water and through altering pore size (Wisniewski *et al.*, 1991). It is possible, therefore, that crosslinking of pectic RGII promotes FT by reducing the opportunities for extracellular ice nucleation and propagation.

A growing number of observations show that the CW undergoes extensive remodelling in response to abiotic stresses (Tenhaken, 2014; Le Gall *et al.*, 2015), suggesting that it may play a role in tolerance to these conditions and recent work indicates the extent of CW modification that occurs during CA (Willick *et al.*, 2018; Takahashi *et al.*, 2019). Pectin methylesterification, a major determinant of calcium crosslinking of pectic homogalacturonan (HG) domains, alters during CA (Solecka *et al.*, 2008; Baldwin *et al.*, 2014) and one recent study indicates that inhibition of pectin methylesterase activity compromises FT (Chen *et al.*, 2018). This is consistent with a positive role for pectin crosslinking in FT. Our results indicate that RGII crosslinking is also important for plant FT, although our data suggest that it plays a role in basal FT rather than the CA response (Fig. 7, Figs S10, S11).

In conclusion, we have demonstrated that fucosylated RGII pectin in the primary CW plays a role in determining plant FT, most likely through B‐dependent dimerization. Future work will determine whether CW pectin crosslinking brings about FT by altering wall elasticity, strength and/or ice nucleation and propagation and whether these effects are specific to RGII or common to all CW components that influence these parameters. Understanding of the mechanistic basis of this phenomenon will allow the identification of further targets for crop improvement.

## Author contributions

HK, MRK, SCF and PEP designed the experiments, analysed the data and wrote the paper; PEP, OK, MS, SJS, GT, IC, RAB, MD, NR and DS conducted the experiments.

## Supporting information


**Fig. S1** Expression of *CBF1‐3* and the CBF target genes *KIN2* and *GOLS3* are all expressed to normal wild‐type levels in *sfr8*.
**Fig. S2** Two insertional mutants for candidate gene At3g50910 fail to show reduced freezing tolerance after cold acclimation.
**Fig. S3** Nucleotide and amino acid sequence of MUR1 showing the SNPs and amino acid substitutions in the mutants *mur1‐1, mur1‐2, mur1‐3 and sfr8*.
**Fig. S4** *sfr8* fails to convert GDP‐mannose to GDP‐fucose.
**Fig. S5** *sfr8* can be complemented by the *MUR1* coding sequence.
**Fig. S6** Cell‐wall fucose content is restored to wild‐type levels in *sfr8* mutants complemented with *MUR1*.
**Fig. S7** Fucose supplementation restores the freezing‐sensitive phenotype of *sfr8* and *mur1‐1* but does not further improve freezing tolerance in wild‐type plants.
**Fig. S8** *mur2* mutants are not impaired in freezing tolerance.
**Fig. S9** The boric acid watering regime restores the WT visible phenotype in *mur1*.
**Fig. S10** *MUR1* is not inducible by low temperature.
**Fig. S11** *sfr8* is more *sensitive‐to‐freezing* than WT even without cold acclimation.
**Table S1** Candidate SNPs identified using Galaxy.

**Table S2** *MUR1* is not upregulated by CBF overexpression.
**Table S3** *MUR1* is not differentially expressed in response to cold acclimation.
Please note: Wiley Blackwell are not responsible for the content or functionality of any Supporting Information supplied by the authors. Any queries (other than missing material) should be directed to the *New Phytologist* Central Office.
