# Supplementary material for: MUR1‐mediated cell‐wall fucosylation is required for freezing tolerance in Arabidopsis thaliana
Source: New Phytol. 2019 Nov 7;224(4):1518–31. doi: 10.1111/nph.16209 (PMC6899859; doi:10.1111/nph.16209)
Supplement: Supplementary file 1 — Fig. S1 Expression of CBF1‐3 and the CBF target genes KIN2 and GOLS3 are all expressed to normal wild‐type levels in sfr8. Fig. S2 Two insertional mutants for candidate gene At3g50910 fail to show reduced freezing tolerance after cold acclimation. Fig. S3 Nucleotide and amino acid sequence of MUR1 showing the SNPs and amino acid substitutions in the mutants mur1‐1, mur1‐2, mur1‐3 and sfr8. Fig. S4 sfr8 fails to convert GDP‐mannose to GDP‐fucose. Fig. S5 sfr8 can be complemented by the MUR1 coding sequence. Fig. S6 Cell‐wall fucose content is restored to wild‐type levels in sfr8 mutants complemented with MUR1. Fig. S7 Fucose supplementation restores the freezing‐sensitive phenotype of sfr8 and mur1‐1 but does not further improve freezing tolerance in wild‐type plants. Fig. S8 mur2 mutants are not impaired in freezing tolerance. Fig. S9 The boric acid watering regime restores the WT visible phenotype in mur1. Fig. S10 MUR1 is not inducible by low temperature. Fig. S11 sfr8 is more sensitive‐to‐freezing than WT even without cold acclimation. Table S1 Candidate SNPs identified using Galaxy. [file NPH-224-1518-s001.pdf]

New Phytologist **Supporting Information**

Article title: **MUR1-mediated cell-wall fucosylation is required for freezing tolerance in *Arabidopsis thaliana***

Authors: Paige E. Panter, Olivia Kent, Maeve Dale, Sarah J. Smith, Mark Skipsey, Glenn Thorlby, Ian Cummins, Nathan Ramsay, Rifat A. Begum, Dayan Sanhueza, Stephen C. Fry, Marc R. Knight and Heather Knight.

Article acceptance date: 28 July 2019

The following Supporting Information is available for this article:

Fig. S1. Expression of *CBF1-3* and the CBF target genes *KIN2* and *GOLS3* are all expressed to normal wild type levels in *sfr8*.

Fig. S2. Two insertional mutants for candidate gene At3g50910 fail to show reduced freezing tolerance after cold acclimation.

Fig. S3. Nucleotide and amino acid sequence of MUR1 showing the SNPs and amino acid substitutions on the mutants *mur1-1, mur1-2, mur1-3* and *sfr8.*

Fig. S4. *sfr8* fails to convert GDP-mannose to GDP-fucose.

Fig. S5. *sfr8* can be complemented by the *MUR1* coding sequence.

Fig. S6. Cell-wall fucose content is restored to wild type levels in *sfr8* mutants complemented with *MUR1*.

Fig. S7. Fucose supplementation restores the freezing-sensitive phenotype of *sfr8 and mur1-1* but does not further improve freezing tolerance in wild type plants.

Fig. S8. *mur2* mutants are not impaired in freezing tolerance.

Fig. S9. The boric acid watering regime restores the WT visible phenotype in *mur1*.

Fig. S10. *MUR1* is not inducible by low temperature.

Fig. S11. *sfr8* is more sensitive to freezing than WT even without cold acclimation.

Table S1. Candidate SNPs identified using Galaxy.

Table S2. *MUR1* is not upregulated by CBF overexpression.

Table S3. *MUR1* is not differentially expressed in response to cold acclimation.

Fig. S1. Expression of *CBF1-3* and the CBF target genes *KIN2* and *GOLS3* are all expressed to normal wild type levels in *sfr8*.

Relative Quantitation (RQ) of transcripts using qRT-PCR in 8-day-old seedlings exposed to cold (5°C) or ambient (20°C) temperature. **(a)** *CBF1-3* transcripts after 2 h of exposure, **(b)** *KIN2* and **(c)** *GOLS3* after 6 h of exposure. Expression is shown after normalisation to *PEX4*. Values were calculated using the ΔΔCT method and the error bars represent RQ_{MIN} and RQ_{MAX} and constitute the acceptable error level for a 95% confidence level according to Student's *t*-test. Error bars indicate the level of variation between technical replicates within one biological replicate experiment. Data are representative of 2 biological replicate experiments. Primers with the following sequences were used to detect transcripts as described previously in Hemsley et al., (2014). *The Plant Cell* **26**: 465-484

CBF1, *CBF2* and *CBF3*: 5'-GCCAAACAAGAAAACCAGGA-3' (forward) and 5'-TCAGCGAAGTTGAGACATGC-3' (reverse). Primers recognise all three transcripts;
KIN2: 5'-CTGGCAAAGCTGAGGAGAAG-3' (forward) and 5'-ACTGCCGCATCCGATATACT-3' (reverse);
GOLS3: 5'-GAGGTTCACAGGCCAAGAAG-3' (forward) and 5'-TCGTTGTAAATGTCCCACCAT-3' (reverse)
PEX4: 5'- TCATAGCATTGATGGCTCATCCT-3' (forward) and 5'-ACCCTCTCACATCACCAGATCTTAG-3' (reverse)

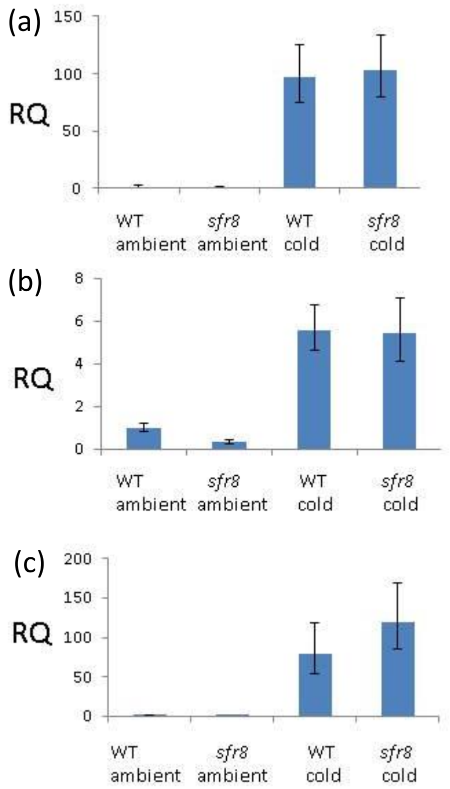

Fig. S2. Two insertional mutants for candidate gene At3g50910 fail to show reduced freezing tolerance after cold acclimation.

(a) Cold-acclimated Col-0 wild type and two insertional mutants one week after a 24 h freezing treatment at -8.5°C.

(b) Both mutants showed reduced levels of At3g50910 transcript compared to wild type. qRT-PCR data showing Relative Quantitation (RQ) of At3g50910 transcripts in the two mutants compared to Col-0 wild type. Error bars present RQ_{MIN} and RQ_{MAX} for three technical replicate measurements as detailed above in the legend for Figure S1.

(a)

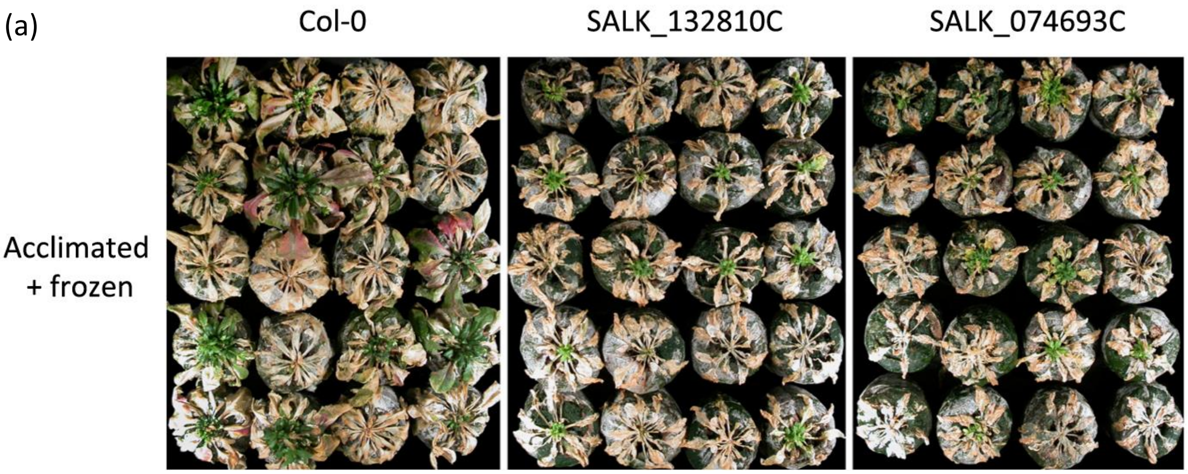

(b)

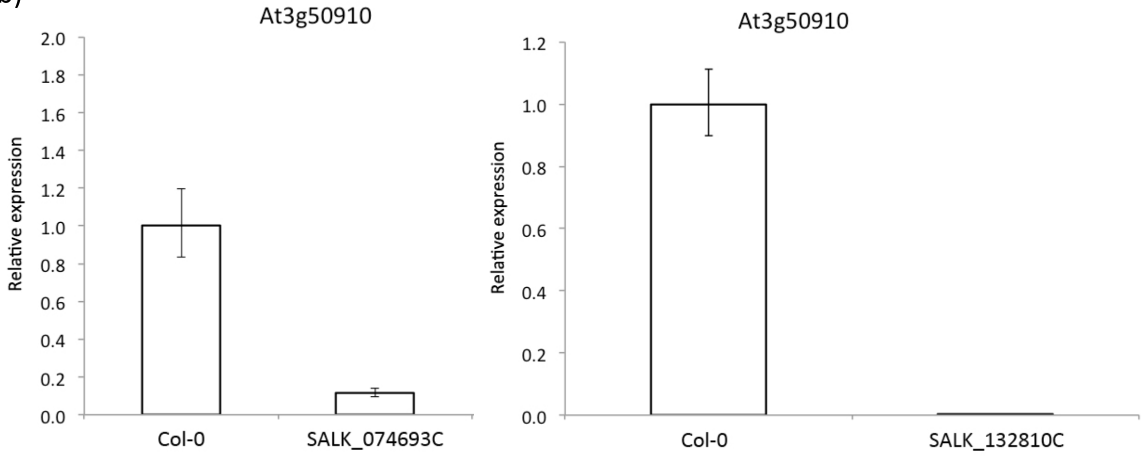

Fig. S3. Nucleotide and amino acid sequence of MUR1 showing the SNPs and amino acid substitutions on the mutants *mur1-1, mur1-2, mur1-3* and *sfr8*.

```
Protein         1  M  A  S  E  N  N  G  S  R  S  D  S  E  S  I  T  A  P  K  A
DNA             1 ATGGCGTCAGAGAACAACGGATCCAGATCCGATTCCGAATCCATCACCGCTCCCAAAGCT

               21  D  S  T  V  V  E  P  R  K  I  A  L  I  T  G  I  T  G  Q  D
               61 GATTCCACCGTCGTTGAACCGAGGAAGATAGCTCTGATCACCGGAATCACCGGCCAGGAC

               41  G  S  Y  L  T  E  F  L  L  G  K  G  Y  E  V  H  G  L  I  R
              121 GGATCATACCTGACGGAGTTCCTTCTCGGAAAAGGCTACGAAGTTCATGGTCTGATCCGT

               61  R  S  S  N  F  N  T  Q  R  I  N  H  I  Y  I  D  P  H  N  V
              181 CGATCATCGAATTTCAACACCCAGCGAATCAACCATATCTACATCGATCCACACAATGTC

               81  N  K  A  L  M  K  L  H  Y  A  D  L  T  D  A  S  S  L  R  R
              241 AACAAAGCTCTGATGAAACTCCACTACGCCGATCTCACCGACGCTTCCTCTCTCCGTCGT

                                     L(mur1-2)
              101  W  I  D  V  I  K  P  D  E  V  Y  N  L  A  A  Q  S  H  V  A
              301 TGGATCGATGTGATCAAACCTGACGAAGTTTATAACCTAGCTGCTCAATCTCACGTCGCT
                                     T

              121  V  S  F  E  I  P  D  Y  T  A  D  V  V  A  T  G  A  L  R  L
              361 GTCTCCTTCGAGATCCCTGATTACACAGCCGATGTAGTCGCAACCGGTGCTCTCCGTCTC

              141  L  E  A  V  R  S  H  T  I  D  S  G  R  T  V  K  Y  Y  Q  A
              421 CTTGAAGCCGTCAGATCTCACACCATCGACAGTGGCCGTACCGTCAAGTATTACCAAGCC

                      F(mur1-1)
              161  G  S  S  E  M  F  G  S  T  P  P  P  Q  S  E  T  T  P  F  H
              481 GGATCTTCGGAGATGTTTGGATCAACTCCTCCTCCACAATCGGAGACGACGCCGTTTCAC
                      T
                                                 V(mur1-3)
              181  P  R  S  P  Y  A  A  S  K  C  A  A  H  W  Y  T  V  N  Y  R
              541 CCCAGATCTCCTTACGCAGCTTCCAAATGCGCTGCTCATTGGTACACAGTGAATTACAGA
                                                 T
                                              E(sfr8)
              201  E  A  Y  G  L  F  A  C  N  G  I  L  F  N  H  E  S  P  R  R
              601 GAGGCGTACGGTCTCTTCGCTTGTAACGGAATCTTGTTCAATCACGAGTCACCTCGCCGT
                                              A

              221  G  E  N  F  V  T  R  K  I  T  R  A  L  G  R  I  K  V  G  L
              661 GGTGAGAATTTCGTGACGAGGAAGATCACAAGAGCATTGGGAAGGATCAAGGTTGGTTTG

              241  Q  T  K  L  F  L  G  N  L  Q  A  S  R  D  W  G  F  A  G  D
              721 CAGACGAAGCTATTCCTTGGGAATTTGCAAGCGTCAAGAGATTGGGGATTTGCAGGAGAT

              261  Y  V  E  A  M  W  L  M  L  Q  Q  E  K  P  D  D  Y  V  V  A
              781 TATGTGGAAGCAATGTGGTTGATGTTGCAGCAAGAGAAGCCAGATGATTACGTTGTGGCA

              281  T  E  E  G  H  T  V  E  E  F  L  D  V  S  F  G  Y  L  G  L
              841 ACAGAGGAAGGACACACAGTGGAAGAGTTTCTTGATGTGTCATTTGGGTATTTGGGACTC

              301  N  W  K  D  Y  V  E  I  D  Q  R  Y  F  R  P  A  E  V  D  N
              901 AATTGGAAAGATTATGTTGAGATTGACCAGAGGTACTTTAGGCCTGCTGAAGTAGATAAC

              321  L  Q  G  D  A  S  K  A  K  E  V  L  G  W  K  P  Q  V  G  F
              961 CTTCAAGGAGATGCAAGCAAGGCAAAGGAAGTGTTGGGGTGGAAACCACAAGTAGGGTTT

              341  E  K  L  V  K  M  M  V  D  E  D  L  E  L  A  K  R  E  K  V
             1021 GAGAAGCTTGTGAAGATGATGGTTGATGAAGATCTTGAGCTTGCTAAGAGGGAGAAAGTG

              361  L  V  D  A  G  Y  M  D  A  K  Q  Q  P  *
             1081 CTTGTTGATGCTGGATACATGGATGCTAAGCAGCAACCTTGA
```

Fig. S4. *sfr8* fails to convert GDP-mannose to GDP-fucose.

The conversion of radiolabelled GDP-mannose to GDP-fucose was assayed using the TLC-based method described by Bonin *et. al.* 1997. Arrows mark the positions of GDP-fucose and GDP-mannose standards. Extracts from wild type (WT) plants and two other freezing sensitive mutants (*sfr4* and *sfr5*) catalysed the conversion of GDP-mannose substrate to GDP-fucose after 70 minutes. Extract from *sfr8* did not.

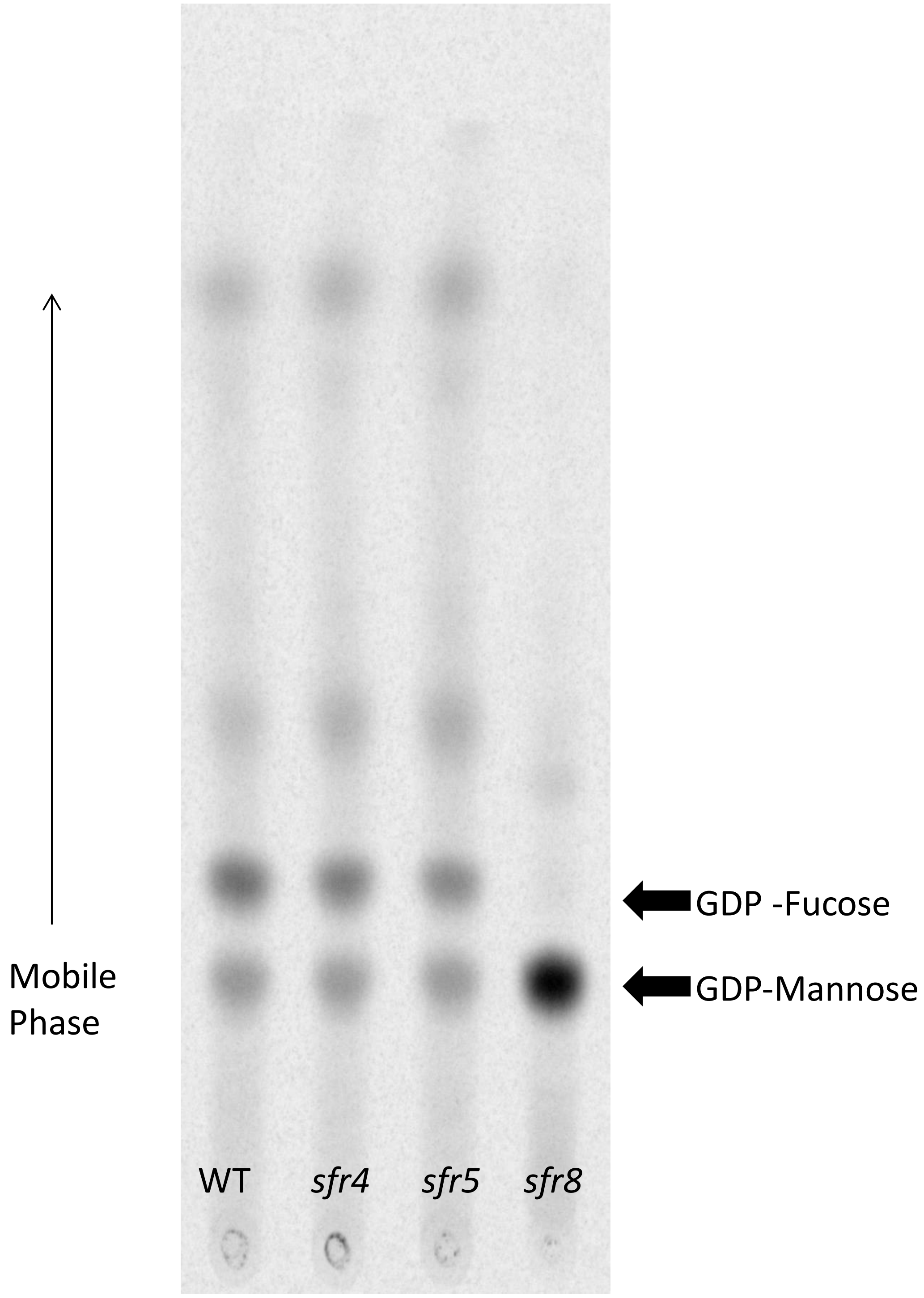

Fig. S5. *sfr8* can be complemented by the *MUR1* coding sequence.

(a) Freeze-induced damage is greater in *sfr8* than wild type but is restored in two additional complemented lines. Percentage electrolyte leakage from leaf discs of Col-0 wild type (WT) plants, *sfr8* and complemented lines 1 (L1) and 8 (L8) after freezing at -6, -8 or -10 °C. Complemented lines 1 and 8, like complemented line 14 (referred to as *sfr8*-C throughout the main text), show restored levels of freezing tolerance. Results from a single experiment are presented; each data point corresponds to the mean of 6 pseudo- biological replicate samples per genotype comprising 3 leaf discs per pseudo-replicate. Error bars represent +/- 1 SEM calculated from arcsine-transformed data.

(b) Cold-acclimated Col-0 wild type (WT), *sfr8*, *mur1-1*, *mur2* and *sfr8* mutant complemented with *MUR1* (complemented line 14*; sfr8-C*) one week after a 24 h freezing treatment at -8.5°C.

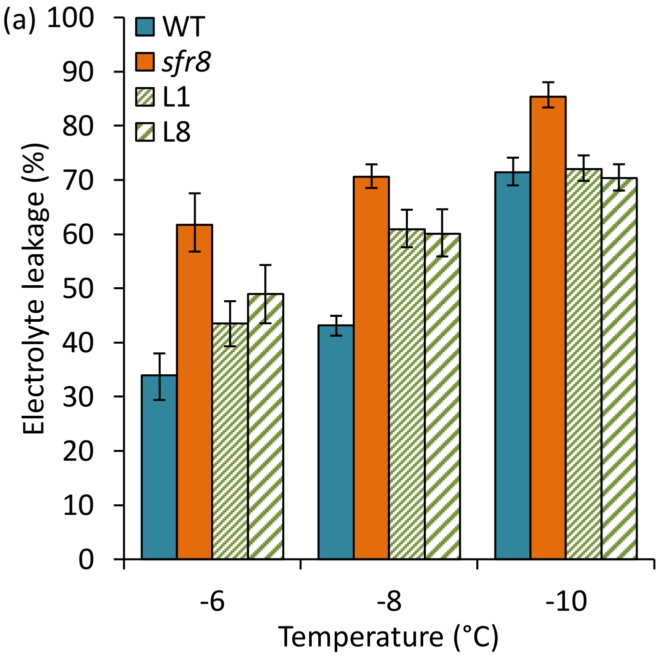

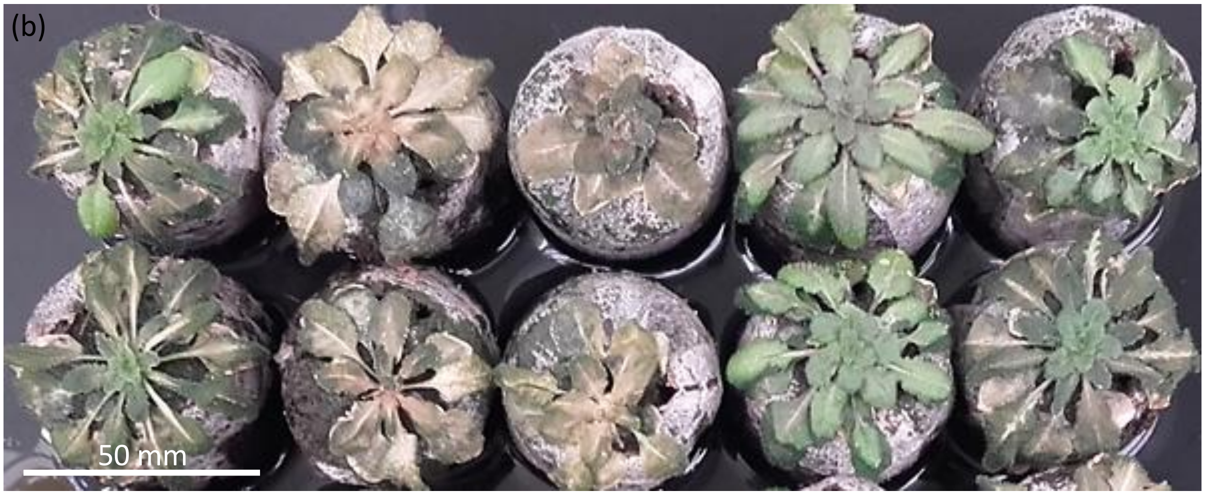

WT *sfr8* *mur1-1* *mur2* *sfr8-C*

Fig. S6. Cell-wall fucose content is restored to wild type levels in *sfr8* mutants complemented with *MUR1*.

Molar percentage (mol %) levels of cell-wall fucose in two-week-old Col-0 wild type (WT), *sfr8* and *sfr8* mutant complemented with the *MUR1* (complemented line 14; *sfr8-C*). Methods are described in the main text and data shown correspond to one biological experiment. Values are means of three measurements and error bars represent +/- SEM.

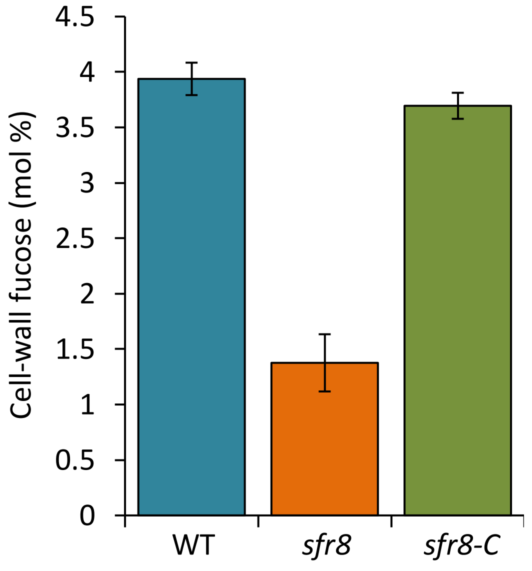

Fig. S7. Fucose supplementation restores the freezing-sensitive phenotype of *sfr8 and mur1-1* but does not further improve freezing tolerance in wild type plants.

Percentage electrolyte leakage values from leaf discs of Col-0 wild type (WT), *sfr8* **(a)** and *mur1-1* **(b)** plants after freezing at -7, -9.5 or -12°C. Plants were grown and cold acclimated as described in the main text and plants sprayed once a week with 60 ml of 10 mM fucose or water during both the growth and cold acclimation periods. Results from a single experiment are presented; each data point corresponds to the mean of 6 pseudo- biological replicate samples per genotype/treatment comprising 3 leaf discs per pseudo-replicate. Error bars represent +/- 1 SEM calculated from arcsine-transformed data.

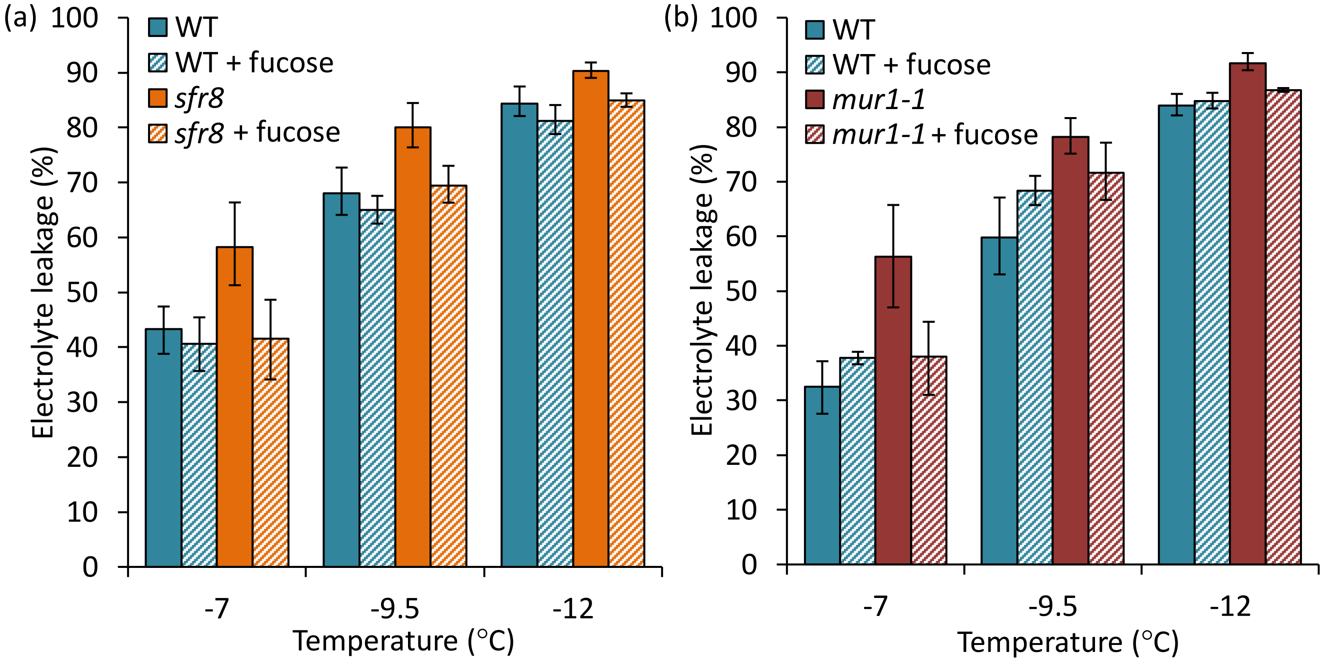

Fig. S8. *mur2* mutants are not impaired in freezing tolerance.

Percentage electrolyte leakage in Col-0 wild type (WT) and *mur2* plants after freezing to -7, -9.5 or -12°C. Plants were grown and cold acclimated as described in the main text. Each data point represents the average of three separate biological replicate experiments. Each experiment used six pseudo- biological replicate samples per genotype comprising three leaf discs per pseudo-replicate. Error bars represent +/- 1 SEM calculated from arcsine-transformed data. Arcsine-transformed percentage leakage data were analysed by a least-squares means comparison at each temperature point (*, $P < 0.05$). A small increase in electrolyte leakage was observed at -7°C only ($P = 0.047$).

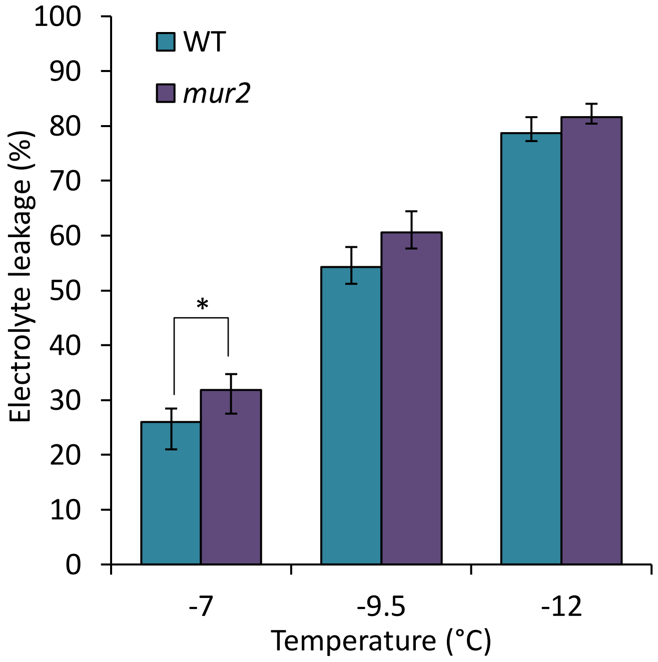

Fig. S9. The boric acid watering regime restores the WT visible phenotype in *mur1*.

Col-0 wild type (WT) and *mur1-1* plants at five weeks old after watering with deionised water (-BA) or 20 mg/l boric acid (+BA). Boric acid (BA)-supplemented *mur1-1* plants show restoration of wild type leaf shape from cup-shaped to spatulate and restoration of petiole length.

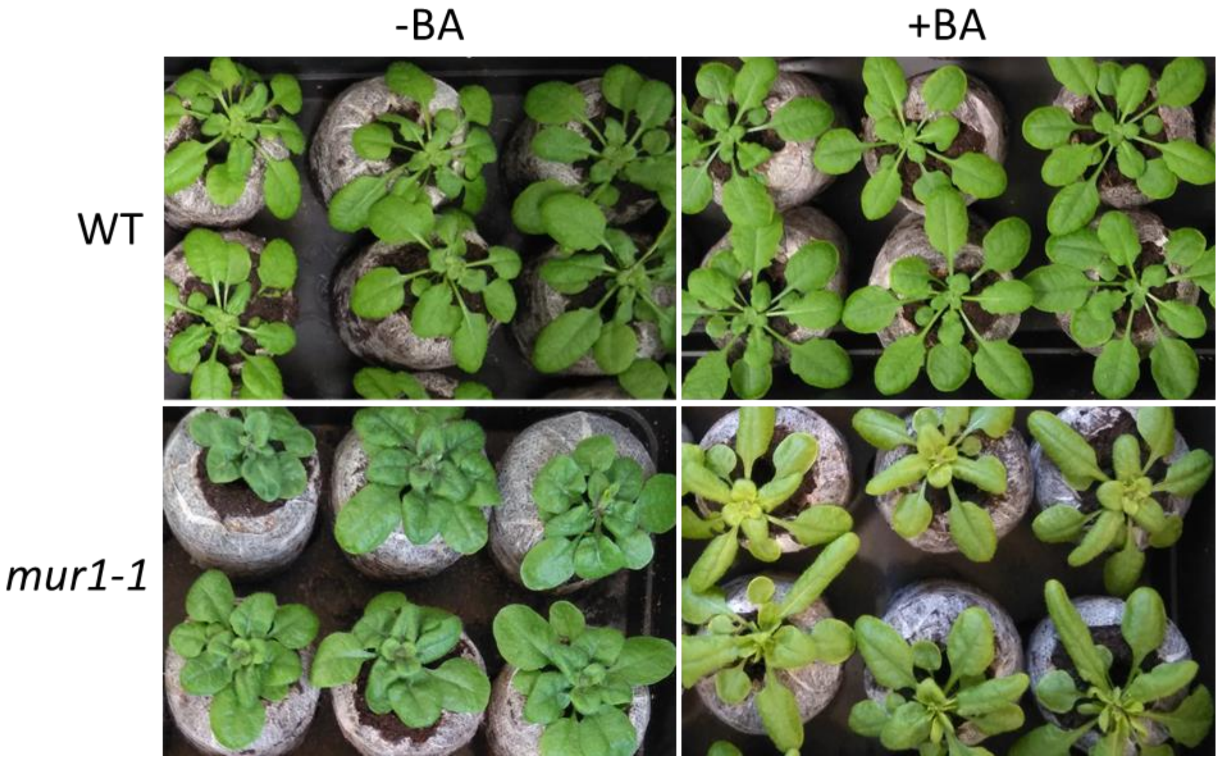

Fig. S10. *MUR1* is not inducible by cold.

(a) Relative quantitation (RQ) by qRT-PCR of *MUR1* and *KIN2* (a known cold-inducible CBF-target gene) transcripts after 1, 3 ,6, 12, 24, 96 or 168 h at 20°C (white bars) or 5°C (grey bars). Expression is shown after normalisation to *PEX4*. Values were calculated using the ΔΔCT method and the error bars represent RQ_{MIN} and RQ_{MAX} and constitute the acceptable error level for a 95% confidence level according to Student's *t*-test. Error bars indicate the level of variation between technical replicates. *KIN2* and *PEX4* primer sequences are shown in Fig. S1; *MUR1* primer sequences were: 5'-ACACCCAGCGAATCAACCAT-3' (forward) and 5'- CGATCCAACGACGGAGAGAG-3' (reverse).

(b) Published data showing that *MUR1* is not significantly differentially regulated in response to cold over a period of 4 days (graph plotted using https://wyguo.shinyapps.io/atrtd2_profile_app/ from Calixto et al. (2018) *The Plant Cell*, 30: 1424-1444) with kind permission from Prof John Brown, The James Hutton Institute, Dundee). Statistical analyses of these data are presented in Table S3 and show no significant differential expression between contrast groups.

(a)

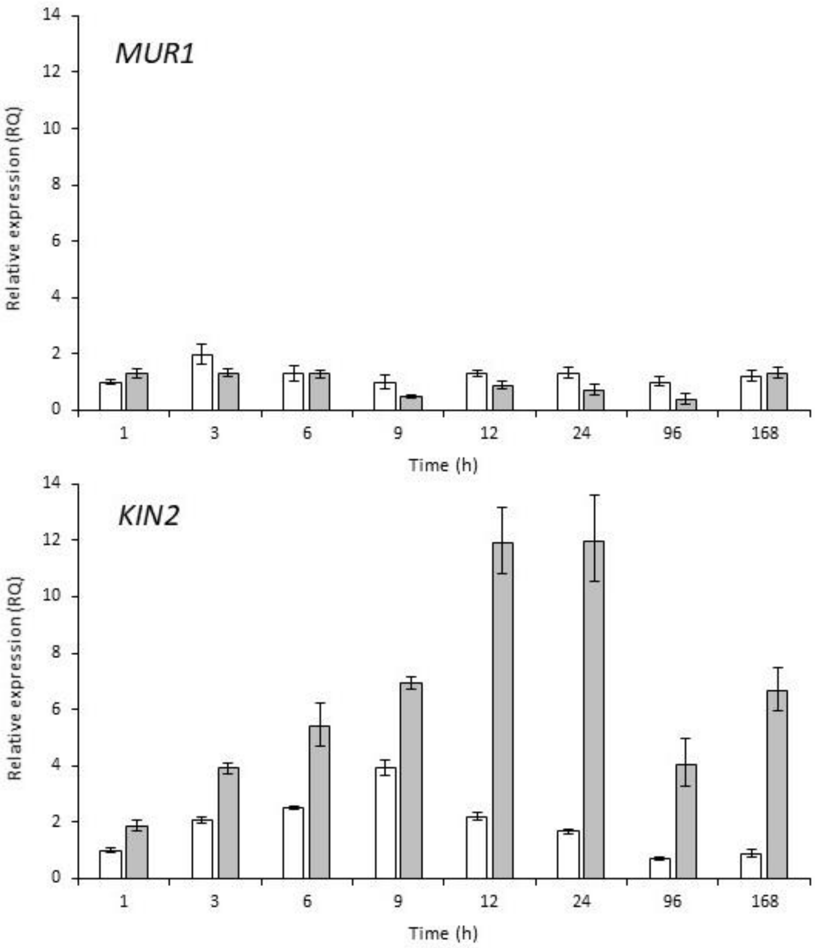

(b)

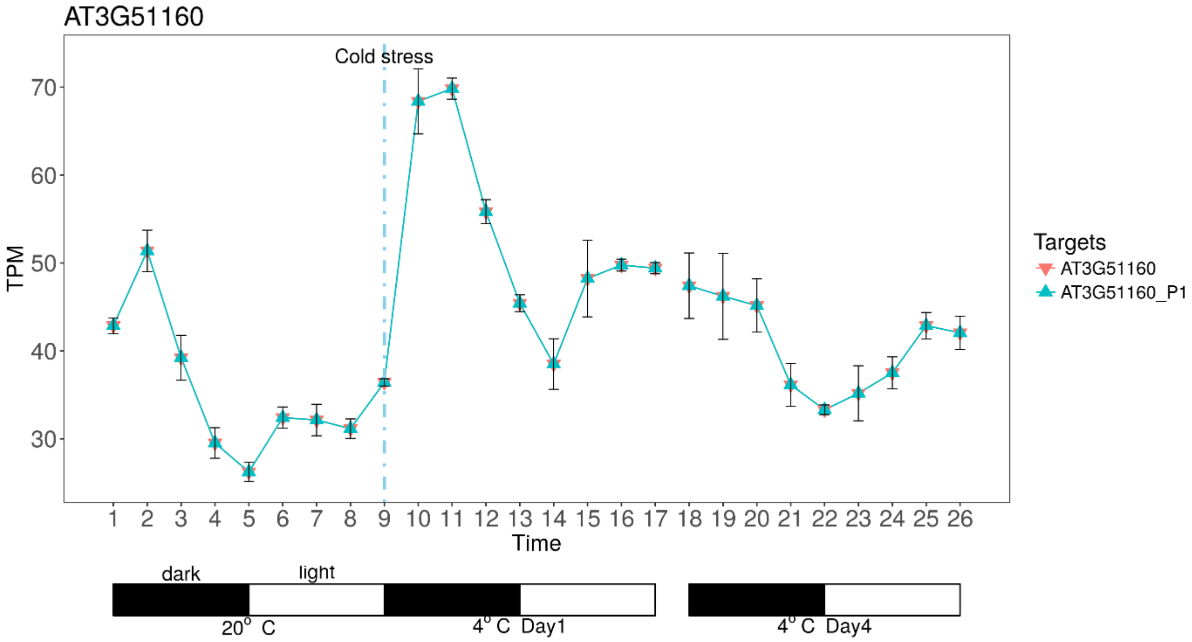
AT3G51160
Cold stress
TPM
70
60
50
40
30
1 2 3 4 5 6 7 8 9 10 11 12 13 14 15 16 17 18 19 20 21 22 23 24 25 26
Time
Targets
AT3G51160
AT3G51160_P1
dark
light
20° C
4° C Day1
4° C Day4

Fig. S11. *sfr8* is more sensitive to freezing than WT even without cold acclimation.

Freeze-induced damage is greater in *sfr8* than wild type even in non-acclimated plants. Percentage electrolyte leakage from leaf discs of Col-0 wild type (WT) plants, *sfr8* after freezing at -2, or -4°C. Plants were grown as described in the main text. Each data point represents the average of two separate biological replicate experiments. Each experiment used six pseudo- biological replicate samples per genotype comprising three leaf discs per pseudo-replicate. Error bars represent +/- 1 SEM calculated from arcsine-transformed data. Arcsine-transformed percentage leakage data were analysed by a least-squares means comparison at each temperature point (***, $P < 0.001$).

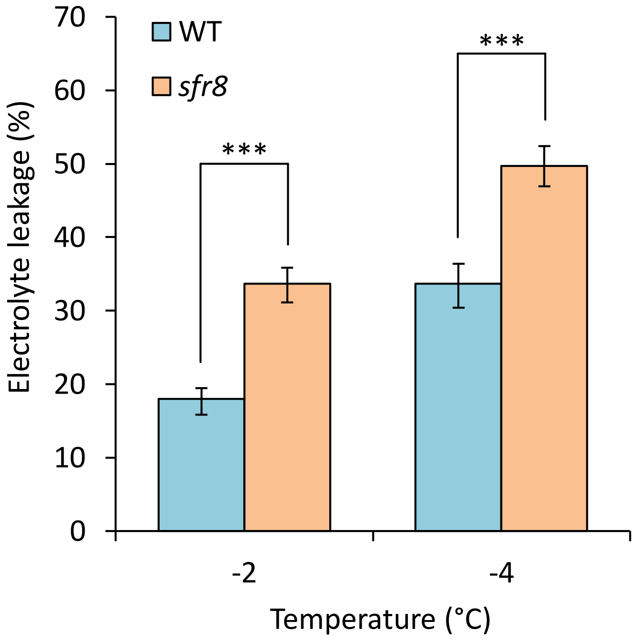

Table S1. Candidate SNPs identified using Galaxy.

Position	Gene	WT base (TAIR10)	*sfr8* sequence base	% of reads showing mutation (total read number)	Confirmation of SNP in *sfr8* genomic DNA
Chr3:18684521	-	T	C	100 (2)	Confirmed but not within a gene.
Chr3:18920586	At3g50910	C	T	100 (16)	Confirmed.
Chr3:19007725	At3g51160	G	A	83 (6)	Confirmed
Chr3:20966136	At3g56590	C	T	100 (25)	Confirmed but within an intron.
